# Distinct migratory pattern of naive and effector T cells through the blood–CSF barrier following Echovirus 30 infection

**DOI:** 10.1186/s12974-019-1626-x

**Published:** 2019-11-21

**Authors:** Marie Wiatr, Carolin Stump-Guthier, Daniela Latorre, Stefanie Uhlig, Christel Weiss, Jorma Ilonen, Britta Engelhardt, Hiroshi Ishikawa, Christian Schwerk, Horst Schroten, Tobias Tenenbaum, Henriette Rudolph

**Affiliations:** 1 Pediatric Infectious Diseases, University Children’s Hospital Mannheim, Medical Faculty Mannheim, Heidelberg University, Theodor-Kutzer-Ufer 1-3, 68167 Mannheim, Germany; 2 Institute for Research in Biomedicine, Università della Svizzera italiana, 6500 Bellinzona, Switzerland; 3 Institute of Microbiology, ETH Zurich, 8093 Zurich, Switzerland; 4 Flowcore Mannheim, Ludolf-Krehl-Strasse 13 – 17, 68167 Mannheim, Germany; 5 Institute of Medical Statistics and Biomathematics, Medical Faculty Mannheim, Heidelberg University, Mannheim, Germany; 6 Immunogenetics Laboratory, Institute of Biomedicine, and Clinical Microbiology, Turku University Hospital, University of Turku, Turku, Finland; 7 Theodor Kocher Institute, University of Bern, Bern, Switzerland; 8 Department of NDU Life Sciences, School of Life Dentistry, Nippon Dental University, Tokyo, Japan

**Keywords:** Enterovirus, Blood–cerebrospinal fluid barrier, T cell migration, Effector T cells, Naive T cells, Meningitis

## Abstract

**Background:**

Echovirus 30 (E-30) is one of the most frequently isolated pathogens in aseptic meningitis worldwide. To gain access to the central nervous system (CNS), E-30 and immune cells have to cross one of the two main barriers of the CNS, the epithelial blood–cerebrospinal fluid barrier (BCSFB) or the endothelial blood–brain barrier (BBB). In an in vitro model of the BCSFB, it has been shown that E-30 can infect human immortalized brain choroid plexus papilloma (HIBCPP) cells.

**Methods:**

In this study we investigated the migration of different T cell subpopulations, naive and effector T cells, through HIBCPP cells during E-30 infection. Effects of E-30 infection and the migration process were evaluated via immunofluorescence and flow cytometry analysis, as well as transepithelial resistance and dextran flux measurement.

**Results:**

Th1 effector cells and enterovirus-specific effector T cells migrated through HIBCPP cells more efficiently than naive CD4^+^ T cells following E-30 infection of HIBCPP cells. Among the different naive T cell populations, CD8^+^ T cells crossed the E-30-infected HIBCPP cell layer in a significantly higher number than CD4^+^ T cells. A large amount of effector T cells also remained attached to the basolateral side of the HIBCPP cells compared with naive T cells. Analysis of HIBCPP barrier function showed significant alteration after E-30 infection and trans- as well as paracellular migration of T cells independent of the respective subpopulation. Morphologic analysis of migrating T cells revealed that a polarized phenotype was induced by the chemokine CXCL12, but reversed to a round phenotype after E-30 infection. Further characterization of migrating Th1 effector cells revealed a downregulation of surface adhesion proteins such as LFA-1 PSGL-1, CD44, and CD49d.

**Conclusion:**

Taken together these results suggest that naive CD8^+^ and Th1 effector cells are highly efficient to migrate through the BCSFB in an inflammatory environment. The T cell phenotype is modified during the migration process through HIBCPP cells.

## Background

Echovirus-30 (E-30) is a non-polio enterovirus (NPEV) responsible for extensive outbreaks of aseptic meningitis in children worldwide [1, 2]. E-30 is a positive-sense single-stranded RNA virus with a high mutation rate belonging to the *Picornaviridae* family [3]. Infections with E-30 result in mild symptoms to lethal outcomes [4, 5]. Patients suffering from severe enterovirus infection frequently require hospitalization, which has a relevant economic impact [6]. Enteroviruses have a broad cell-tropism and can infect a wide range of cells such as neurons, cardiomyocytes, and epithelial cells [7].

Clinical studies revealed that enterovirus infection of the central nervous system (CNS) resulted in increased levels of inflammatory cytokines within the cerebrospinal fluid (CSF), such as INF-γ, IL-6, and CXCL12 [8, 9], accompanied by influx of neutrophils and T cells [4]. At the beginning of NPEV-caused meningitis, an abundant concentration of polymorphonuclear neutrophils (PMN) and T cells were detected in the CSF of patients [10], especially IFN-γ-producing Th1 cells can be found in the course of disease [11]. Their secretion of high levels of INF-γ enhances the activation of other immune cells such as macrophages and dendritic cells. In the resolving phase of the disease, a switch from Th1 to Th2 producing IL-4, IL-5, and IL-13 cells is observed [12].

During meningitis caused by E-30 infection, the virus can potentially interact with the two main CNS barriers, the blood–brain barrier (BBB) and the blood–CSF barrier (BCSFB) [13, 14]. The BCSFB is located at the choroid plexus in the ventricles of the brain [15]. It consists of epithelial cells connected by tight and adherens junctions [16] and is responsible for the production and secretion of the majority of the CSF [17, 18]. In a recent review culture models to study leukocyte trafficking though the BCSFB were extensively described [19]. In an in vitro model of the BCSFB based on human immortalized brain choroid plexus papilloma (HIBCPP) cells, it has been shown that HIBCPP cells can be infected with human enterovirus, such as E-30 [20, 21]. The infection can cause a barrier alteration accompanied by a drop of the transepithelial electrical resistance (TEER), thus possibly promoting invasion of pathogens and leukocytes through HIBCPP cell layers. Moreover, it was shown that infection of this BCSFB in vitro model resulted in inflammatory cytokine release such as IL-6 and CCL20, creating a pro-inflammatory environment leading to migration of immune cells, such as neutrophils or T cells, through the choroid plexus epithelium [22, 23]. In contrast, in healthy individuals there is a low but continuous trafficking of immune cells through the blood–brain barriers into the brain [24, 25].

A differentiated analysis of naive and T effector cell migration across the choroid plexus and additionally in the context of CNS infection has not been performed before. In this study, we compared the migration of naive and effector CD4^+^ and CD8^+^ T cells across a human in vitro model of the BCSFB based on HIBCPP cells following E-30 infection. We further identified the migration pathways of T cells crossing HIBCPP cells. Finally, we characterized the T cell phenotype migrating through HIBCPP cells.

## Material and methods

### Human immortalized brain choroid plexus papilloma cell culture and evaluation of barrier integrity

HIBCPP cells were previously characterized [26] and derived from the right lateral ventricle tumor of a 29-year-old Japanese woman who underwent intracranial tumor resection. HIBCPP cells were cultured on cell culture inserts (Millipore, Germany; pore diameter 5.0 μm, pore density 6.0 × 10^5^ pores/cm^2^, 0.33 cm^2^) in the inverted culture model in DMEM/HAM’s F12 1:1 medium supplemented with 4 mM l-glutamine, 5 μg/ml insulin, 10% fetal calf serum (FCS) on 24-well plates. When the cells reached a TEER > 70 Ω cm^2^, they were switched to DMEM/HAM’s F12 1:1 medium supplemented with 4 mM l-Glutamine, 5 μg/ml insulin, 1% heat-inactivated fetal calf serum (FCS) 1% for 24 h prior to performing the experiment. Barrier integrity was evaluated via the measurement of (TEER) with a tissue voltohmmeter (Millipore, Germany) at 0 h, 24 h, and 28 h [26]. The paracellular permeability was determined as published earlier with Dextran-TexasRed© (Invitrogen, Germany) tracer solution (1000 MW) [20]. In brief, Dextran-TexasRed© (Ex595/Em615) (1000 MW) tracer solution was added at 24 h post infection to the basolateral side of the HIBCPP cell layer, and the paracellular permeability was evaluated 28 h post infection via fluorescence measurement with a TECAN 200 M Infinite Multiwell reader (Tecan, Männedorf, Switzerland).

### Isolation of naive T cells

Blood from healthy donors was processed with Biocoll (Merck, Germany) following the manufacturer’s instructions to isolate PBMC. PBMCs were kept in culture for 24 h in RPMI 1640 medium (Merck, Germany) supplemented with 10% FCS at 37 °C, 5% CO_2_. Isolation of CD3^+^, CD4^+^, and CD8^+^ lymphocytes was performed using Invitrogen Dynabeads© negative isolation kit selection according to the manufacturer’s instructions (Thermofischer, USA), and resulted in a purity of 90–95% for the three cell types. Isolated cell were characterized as mainly naive cells by flow cytometry analysis showing high expression of CD62L (L-selectin) and CD45RA, and low expression of CD45RO and CD69.

Approval for blood draw was obtained by the local ethics committee of the Medical Faculty of Mannheim, Heidelberg University (2009-327 N-MA).

### Th1 cell selection culture and expansion

Human Th1 cells were isolated by flow cytometry to their specific expression pattern of chemokine receptors (CXCR3^+^CCR4^−^CCR6^−^) out of CD4^+^CD45RA^−^ T cells from PBMCs of healthy donors as previously published [27–29] (Additional file 1) at the Institute for Research in Biomedicine (Bellinzona, Switzerland). This cell population is stable and further characterized with intracellular cytokine staining. It revealed, as expected, a high expression of IL-4 and IFNγ (Additional file 2). Th1 effector cells were stimulated with PHA, allogenic irradiated PBMCs, and RPMI medium (Merck, Germany) supplemented with 10% heat-inactivated FCS and IL-2 (500 IU/ml). After 20 days, cells were analyzed for chemokine receptor and intracellular cytokine expression before cells were frozen and kept in liquid nitrogen until further use. After thawing, Th1 cells were further expanded for 21 days in RPMI medium (Merck, Germany) supplemented with 10% FCS and IL-2 (500 IU/ml) in 24-well plates (CytoOne, Hamburg).

Approval was obtained by the Swiss Federal Office of Public Health (authorization no. A000197/2 to F.S.).

### Culture of Coxsackievirus B4–specific T cells

We received Coxsackievirus B4 (CVB4)–specific T cells from Dr. Ilonen of the Institute of Biomedicine, University of Turku, Turku, Finland. CVB4-specific T cells were obtained as described previously. In brief, PBMCs were isolated via Ficoll gradient solution and further incubated with CVB4 antigen (10μg/ml) supplemented with 10% human AB serum, gentamicin sulfate (10μg/ml), HEPES buffer solution, 1 M (20μl/ml), and 3% glutamine in RPMI 1640 medium. Further, CD8^+^ cells were depleted and only the CD4^+^ CVB4-specific cells were kept. After 7 days, fresh medium supplemented with IL-2 (20 U/ml) was added. After 14 days in culture, the T cells were restimulated with both CVB4 antigen and irradiated (30 Gy) autologous antigen-presenting cells (2 × 10^6^ PBMC/ml) (for further information please refer to [30]). The CVB4-specific T cells were thawed and cultivated in RPMI 1640 medium (Merck, Germany) supplemented with 10% FCS 2 days before being used for experiments.

### Virus preparation

Echovirus 30 (E-30) strain *Bastianni* (further called E-30 within the manuscript) was kindly provided by the National Reference Center for Poliomyelitis and Enteroviruses (NRC PE), at the RKI (Berlin, Germany) and propagated as published earlier [20]. In brief, E-30 was propagated using confluent RD cells (Rhabdomyosarcoma cells). When a cytopathic effect of 90% was reached, the virus-cell suspension was frozen over night at − 20 °C. The suspension was further centrifuged at 4000 rpm 15 min at 4 °C. The aliquoted were frozen at − 80 °C. The number of viral copies was determined via Quantitative TaqMan real-time PCR analysis [20].

### Infection of the HIBCPP cells with E-30 *Bastianni*

**Fig. 1:**
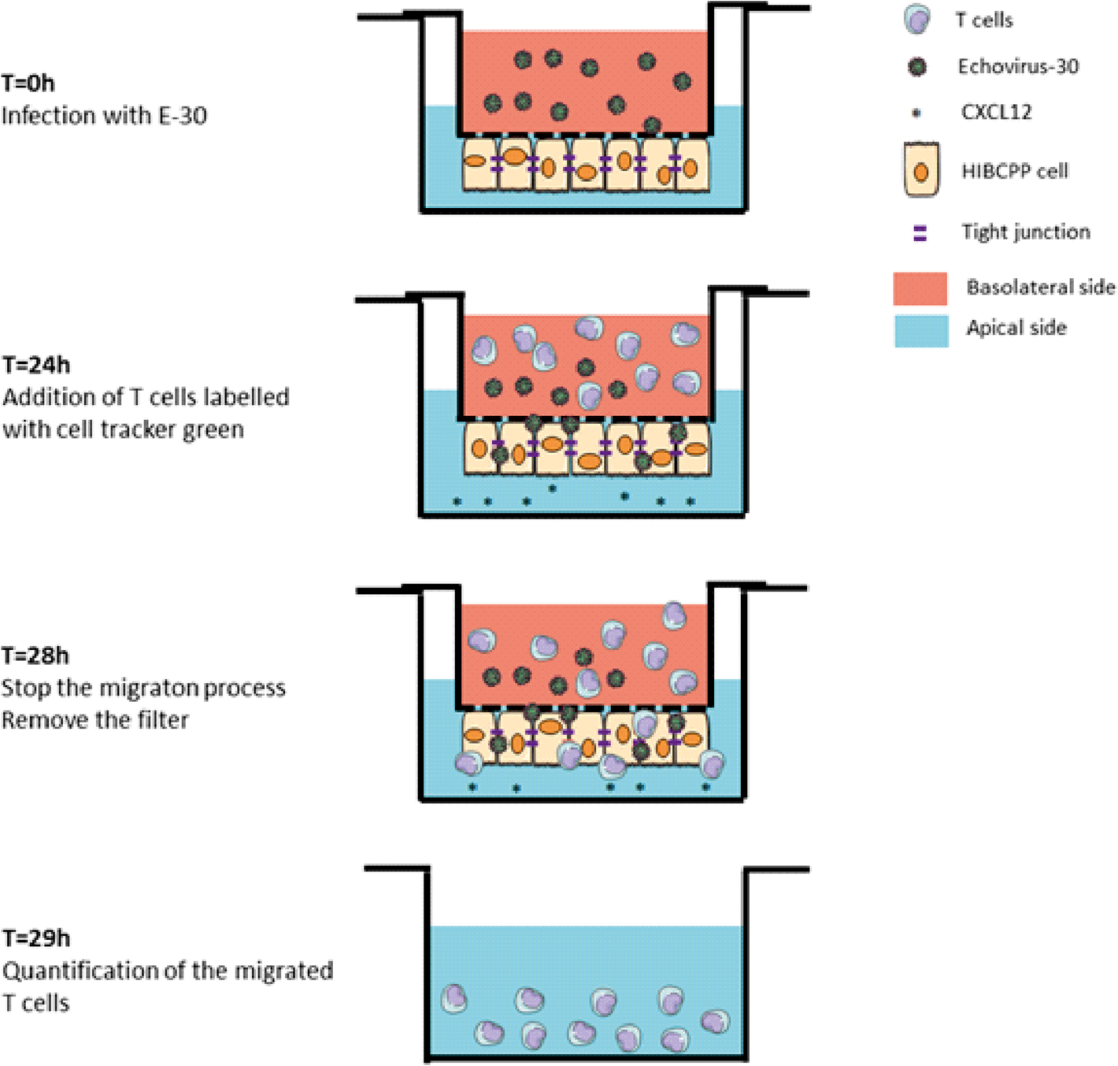
Schematic image of the experimental set-up of transmigration experiments. At first HIBCPP cells were infected with E-30 MOI (0.7) T = 0 h. At T = 24 h post infection T cells were labeled with cell tracker green and added to the upper compartment of the well. CXCL12 was added to the bottom of the well in some conditions. At the end of the 4 h of migration experiment the filters were removed and the T cells present on the bottom part of the well were counted under the microscope

Once HIBCPP cells grown on cell culture inserts in the inverted cell culture model reached TEER values between 220 and 670 Ω cm^2^, cells were used for experiments. All experiments were performed in migration assay medium (MAM) consisting of RPMI 1640 medium containing 5% FCS supplemented with 2% glutamine and 25 mM HEPES. To the upper compartment of the filter, 400 μl of MAM containing E-30 at a multiplicity of infection (MOI) of 0.7 was added and kept throughout the experiment (28 h). As previously shown the stimulation of HIBCPP with a MOI of 0.7 led to an effective infection and replication ([21], Additional file 3). For uninfected controls, only MAM was added to the upper compartment. A schematic representation of the experimental set-up is displayed in Fig. 1.

### Migration assay of T cells and quantification of migrating T cells

CD3^+^, CD8^+^, CD4^+^, Th1, or CVB4-specific T cells were labeled with cell tracker green (Life Technologies, USA) following the manufacturer’s instructions. T cells at a concentration of 4 × 10^5^ were added to the upper compartment of the insert facing the basolateral side of HIBCPP cells following 24 h of infection with E-30 at MOI 0.7. To the bottom of the plates (apical cell side), 1000 μl of MAM ± CXCL12 (200 ng/ml; Preprotech, Germany) was added for control conditions, since CXCL12 has been demonstrated to be an important chemokine in the CSF also in the context of neuroinflammation [9]. At the end of the 4 h of migration, the layers of HIBCPP cells were rinsed two times in PBS, fixed in 3.7% formaldehyde and stored for further immunofluorescence. The number of migrated T cells was evaluated microscopically (Zeiss Apotome, Germany) via counting of cells present on the bottom of the well. Ten fields of views were taken randomly in the well with a Zeiss Apotome and Zen software (Carl Zeiss, Germany) using an X10/1.4 objective lens. Quantification of the number of T cells was performed using Image J software.

### Immunofluorescence

At the end of the migration assays, HIBCPP cells were rinsed in PBS (Gibco, Thermofischer USA), and fixed in 3.7% formaldehyde for 15 min at room temperature (RT). HIBCPP cells were further washed in PBS, and culture inserts were cut out and permeabilized for 20 min with 1% Triton-X-100 PBS in 1% BSA at RT. Blocking was performed by incubating the cells in 1% BSA/PBS solution for 15 min at RT. As primary antibody monoclonal mouse light diagnostics™ anti-PAN Enterovirus (1:250; Merck, Germany) was used overnight at 4 °C. Cells were washed in PBS and incubated with the secondary antibody Alexa Fluor 594 goat anti-mouse, and simultaneously with phalloidin Alexa Fluor 660 (1:250 Molecular Probes, USA) and 4′-6-diamidino-2-phenylindole dihydrochloride (DAPI) (concentration 1:50,000) for 1 h at RT, respectively. Cells were washed in PBS and mounted with antifade reagent (Life Technologies, USA). The quantification of T cells associated with HIBCPP cell layer was performed by counting the number of T cells present in 10 fields of views, which were selected randomly. Images were taken with a Zeiss Apotome and Zen software (Carl Zeiss, Germany) using an X63/1.4 objective lens. For evaluation of the migration pathway of the T cells, z-stacks from cell layers were acquired using Zeiss Apotome and Zen software (Carl Zeiss, Germany) using X63/1.4 objective lens.

### Flow cytometry

Flow cytometry analysis was performed on Th1 effector cells, which had either migrated, or not, across HIBCPP cell layers. The non-migrated effector T cells in the upper compartment in uninfected controls or in the E-30-infected condition, as well as the cells within the lower compartment in uninfected condition, were collected separately. Filter compartments of the same condition were pooled together (*n* = 36, each). Th1 effector T cell single cell selection for the flow cytometry analysis is displayed in Additional file 4. Next, the cells were rinsed twice in 1% PBS/FCS to be further centrifuged 8 min at 1600 rpm. Th1 effector cells with no contact to HIBCPP cells in uninfected condition (UI) and Th1 effector cells with no contact of HIBCPP cells in infected condition were used as controls. The viability of Th1 effector cells was assessed via fixable viability dye eFluor™ 780 following the manufacturer’s instructions (ThermoFisher Scientific, Germany). Th1 effector cells were washed twice with PBS and fixed with 3.7% formaldehyde. Hereafter, Th1 effector cells were washed twice in PBS prior to the staining and incubated with a Human BD Fc Block™ (BD Biosciences, Germany) to avoid unspecific binding during the staining. Further, Th1 effector cells were stained with two different antibody panels (panel 1 or panel 2; see also Table 1) following the manufacturer’s instructions. After 30 min of incubation at 4 °C in the dark, Th1 effector cells were rinsed twice in PBS and kept at 4 °C for analysis. Flow cytometry analysis was conducted on the FACS Aria I PMT machine in the Flowcore Institute, Mannheim. Results were processed using Flowjo™ software 7.6.5.

**Table 1:** Antibodies used for the flow cytometry analysis

	Color	Company	Isotype	Reference
Mix 1 antibodies				
CXCR3	APC	BD	Mouse IgG1 κ	561732
CCR4	PE	BD	Mouse IgG1 κ	561110
CCR6	FITC	Biolegend	Mouse IgG2b κ	353411
CD44	BV510	BD	Mouse IgG2b κ	BD563029
PSGL-1	BV421	BD	Mouse IgG1 κ	BD743478
CD62L	PE-Cy7	BD	Mouse IgG1 κ	565535
Mix 2 antibodies				
LFA-1	APC	BD	Mouse IgG1 κ	BD551060
CCR7	PE	Biolegend	Mouse IgG2a κ	FAB197P025
CCR5	FITC	BD	Mouse IgG2a κ	561747
Integrin β7	BV510	Biolegend	Mouse IgG1 κ	304314
CD29	BV421	BD	Mouse IgG1 κ	BD563514

For T cell characterization, flow cytometry was performed for migrated versus non-migrated T cells. The antibodies displayed within the table were used. The following antibodies were grouped as CXCR3-APC, CCR4-PE, CCR6-FITC, CD44-BV510, PSGL-1-BV421, CD62-L-PE-Cy7 for Mix 1 and LFA-1- β1-APC, CCR7-PE, CCR5-FITC, CD49d-PE-Cy7, CD29-BV510, and β7-BV421 for Mix2. For both mixes live/dead-APC-Cy7 was present

### Statistical analysis

Statistical analyses were conducted using SAS Software, release 9.4 (SAS Institute Inc., Cary, NC, USA). ANOVAs for repeated measurements have been performed with the SAS procedure PROC MIXED. The condition (UI, UI + CXCl12, E-30 and E-30 + CXCL12) has been considered a fixed factor whereas the number of experiments has been handled as a random factor. Furthermore, Tukey–Kramer tests have been performed for pairwise comparisons. The figures are represented as mean ± SD. Each of the 4 conditions (UI, UI + CXCL12, E-30 and E-30 + CXCL12) was considered a fixed factor.

## Results

### Increased migration of Th1 effector cells compared with naive T cells through HIBCPP cells after E-30 infection

Previous studies have shown the capacity of naive CD3^+^ T cells and PMN to migrate across the in vitro model of HIBCPP cells after E-30 infection [31]. Now, we compared the migration capacity of different subpopulations of naive T cells, (CD3^+^, CD4^+^, and CD8^+^ T cells) in presence or absence of the chemokine CXCL12 and/or E-30 infection. When HIBCPP cells grown to confluence on filter inserts they reached a high TEER and then were infected with E-30 at a MOI of 0.7 on their basolateral side (upper part of the filter). The different types of T cells were further added on the basolateral side of the HIBCPP cells following 24 h of infection with E-30. CXCL12, a CSF chemokine, which is upregulated during neuroinflammation and is known to promote leukocyte migration, was added or not to the apical side of HIBCPP cells (Fig. 1).

**Fig. 2:**
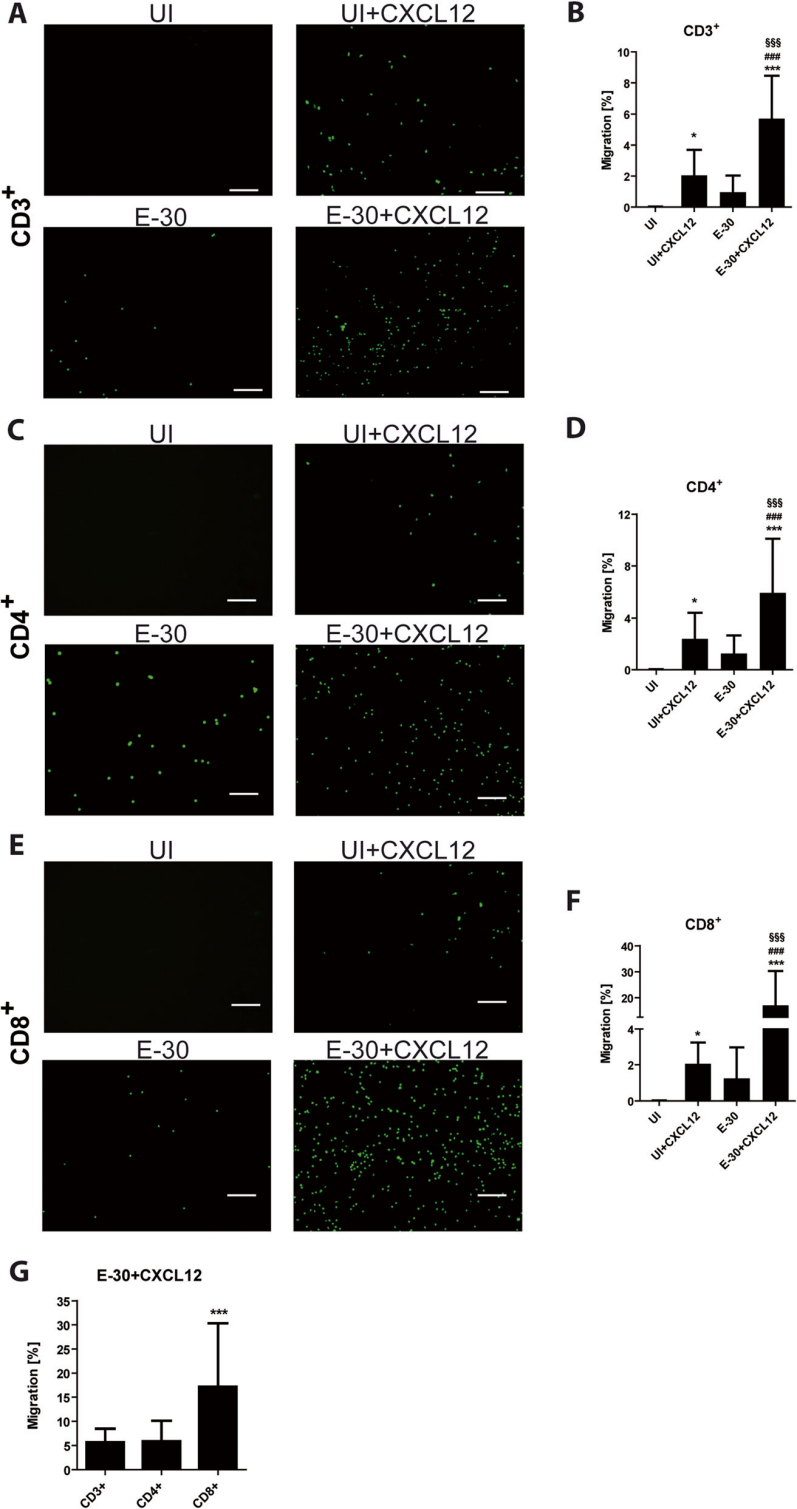
E-30 infection of HIBCPP cells enhances migration of naive T cells. Migration of naive CD3^+^, CD4^+^, CD8^+^ T cells across HIBCPP cells was evaluated via immunofluorescence imaging in the following conditions: UI, UI + CXCL12, E-30 and E-30 + CXCL12. T cells were stained with cell tracker green Alexa 488. After 4 h of migration across HIBCPP cells immunofluorescence imaging (10 FOV per condition) was taken for CD3^+^ (**a**), CD4^+^ (**c**), and CD8^+^ (**e**) T cells present in the bottom well. **b**, **d**, **f** represent quantifications of T cells present in the bottom well after 4 h of migration for CD3^+^ CD4^+^ and CD8^+^ respectively. **g** Shows the quantification of naive T cells in E-30 + CXCL12 condition. The data are shown as mean ± SD of 6 independent experiments each performed in triplicate. Statistical significance was calculated using a Tukey–Kramer test. *p* values are displayed as follows: *p < 0.05 and ***p < 0.0001. *p* < 0.05 (*) was reached comparing UI to UI + CXCL12; *p* < 0.0001 (***) was reached comparing UI to E-30 + CXCL12 and comparing CD8^+^ with CD3^+^ and CD4^+^ after E-30 + CXCL12 stimulation; *p* < 0.0001 (###) was reached comparing UI + CXCL12 to E-30 + CXCL12, and *p* < 0.0001 (§§§) was reached comparing E-30 to E-30 + CXCL12. The white scale bar represents 100 μm

**Fig. 3:**
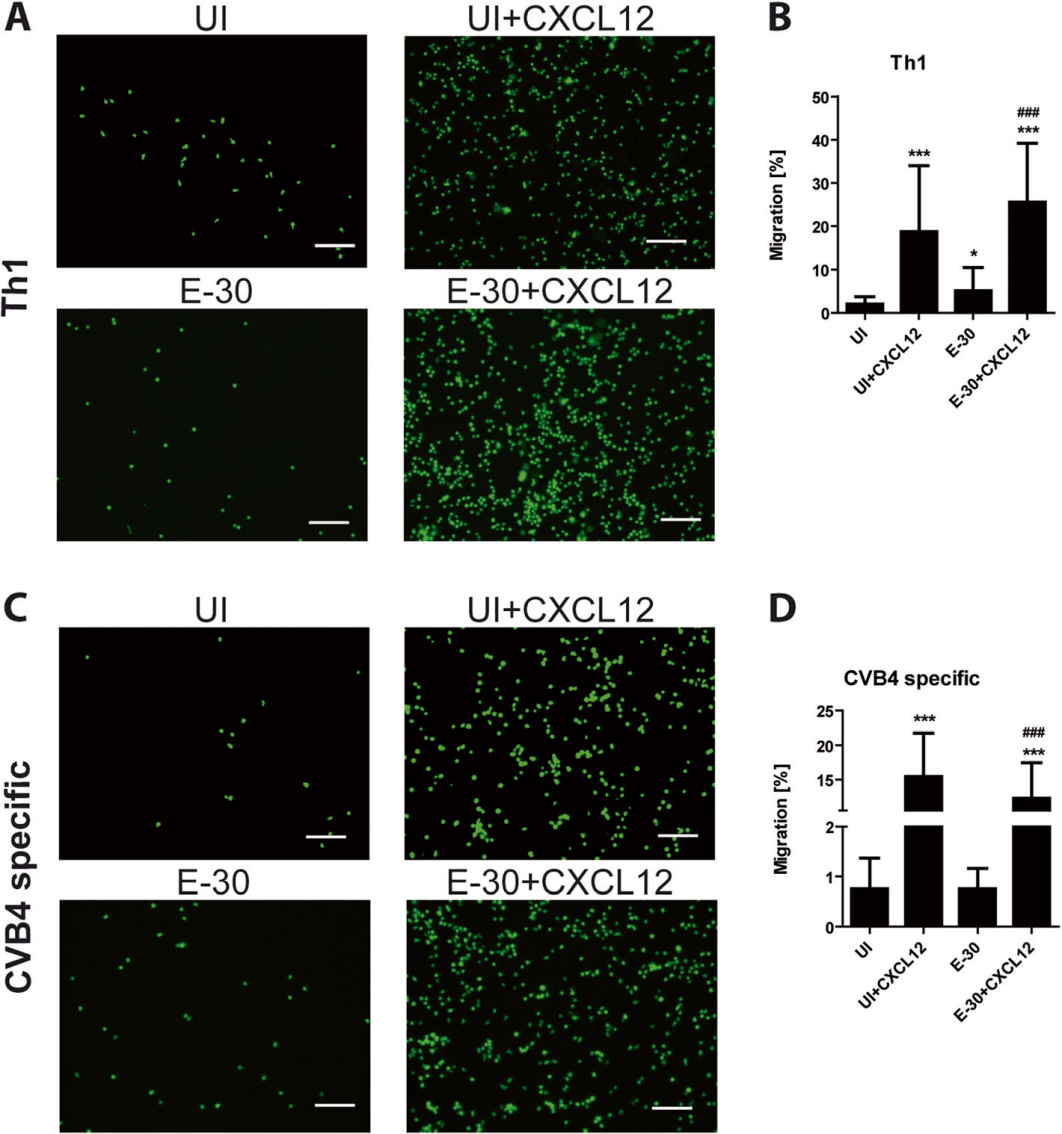
E-30 infection of HIBCPP cells enhances migration of effector T cells. Migration of Th1 effector and CVB4-specific T cells across HIBCPP cells was evaluated via immunofluorescence imaging in the following conditions: UI, UI + CXCL12, E-30 and E-30 + CXCL12. T cells were stained in cell tracker green. Immunofluorescence imaging (10 FOV per conditions) of Th1 (**a**) and CVB4-specific T cells (**c**) present in the bottom well was taken after 4 h of migration across HIBCPP cells for every condition. **b**, **d** Quantification of Th1 (**b**) and CVB4-specific T cells (**d**) present in the bottom well after 4 h of migration for all the conditions. The data are shown as mean ± SD of 6 independent experiments each performed in triplicate. Statistical significance was calculated using a Tukey–Kramer test. *p* values are displayed as follows: *p < 0.05 and *** *p* < 0.0001. *p < 0.05 was reached comparing UI to E-30; *p* < 0.0001 (***) was reached comparing UI to UI + CXCL12, and UI to E-30 + CXCL12; *p* < 0.0001 (###) was reached comparing E-30 against E-30 + CXCL12. The white scale bar represents 100 μm

In the presence of apical CXCL12 and after additional E-30 infection all T cell subpopulations displayed significant higher migration rates compared with the uninfected control conditions (Fig. 2b, d, f and Fig. 3c–d). When CXCL12 was present on the apical side of HIBCPP cells, the migration rates of naive T cells were for CD3^+^ to 1.87 ± 1.60%, for CD4^+^ to 2.45 ± 1.98%, and for CD8^+^ to 2.54 ± 1.65% each (*p* < 0.05) (Fig. 2a–f). Moreover, the naive T cells showed a significantly enhanced migration following E-30 infection + CXCL12 compared with the condition without infection (Fig. 2b, d, f). Following E-30 infection naive CD8^+^ T cells had a significantly higher migration rate of 17.12 ± 12.02% compared with naive CD3^+^ T cells with 5.89 ± 2.62 (*p* = 0.0017) and naive CD4^+^ T cells with 6.04 ± 3.74% (*p* = 0.0004) in the presence of CXCL12 (Fig. 2g).

We further analyzed the migration capacity of effector T cells (Th1 and CVB4-specific T cells) in the same experimental setup as described above and compared it to naive T cell migration. We observed that in the absence of E-30 infection and no stimulation with apical CXCL12, nearly no CD3^+^, CD4^+^, or CD8^+^ T cells crossed HIBCPP cells (Fig. 2a–f), whereas 1.53 ± 1.42% of CD4^+^ Th1 effector cells and 0.75 ± 0.61% of CVB4-specific T cells (both CD4^+^ T cells) migrated through HIBCPP cells (Fig. 3a–d). The migratory potential towards CXCL12 was significantly higher compared with the condition without chemokine: Th1 21.20 ± 16.22% (*p* < 0.0001), and CVB4-specific T cells 15.47 ± 6.26% (*p* < 0.0001) (Fig. 3). Th1 effector cells had also a tendency to migrate more when HIBCPP cells were infected with E-30 compared with the uninfected control condition (3.37 ± 2.75%) (Fig. 3a–b). In contrast, E-30 infection of HIBCPP cells had no impact on the migration of CVB4-specific T cells (Fig. 3c–d).

### Th1 and CVB4-specific T cells adhere to the basolateral side of the HIBCPP cells

**Fig. 4:**
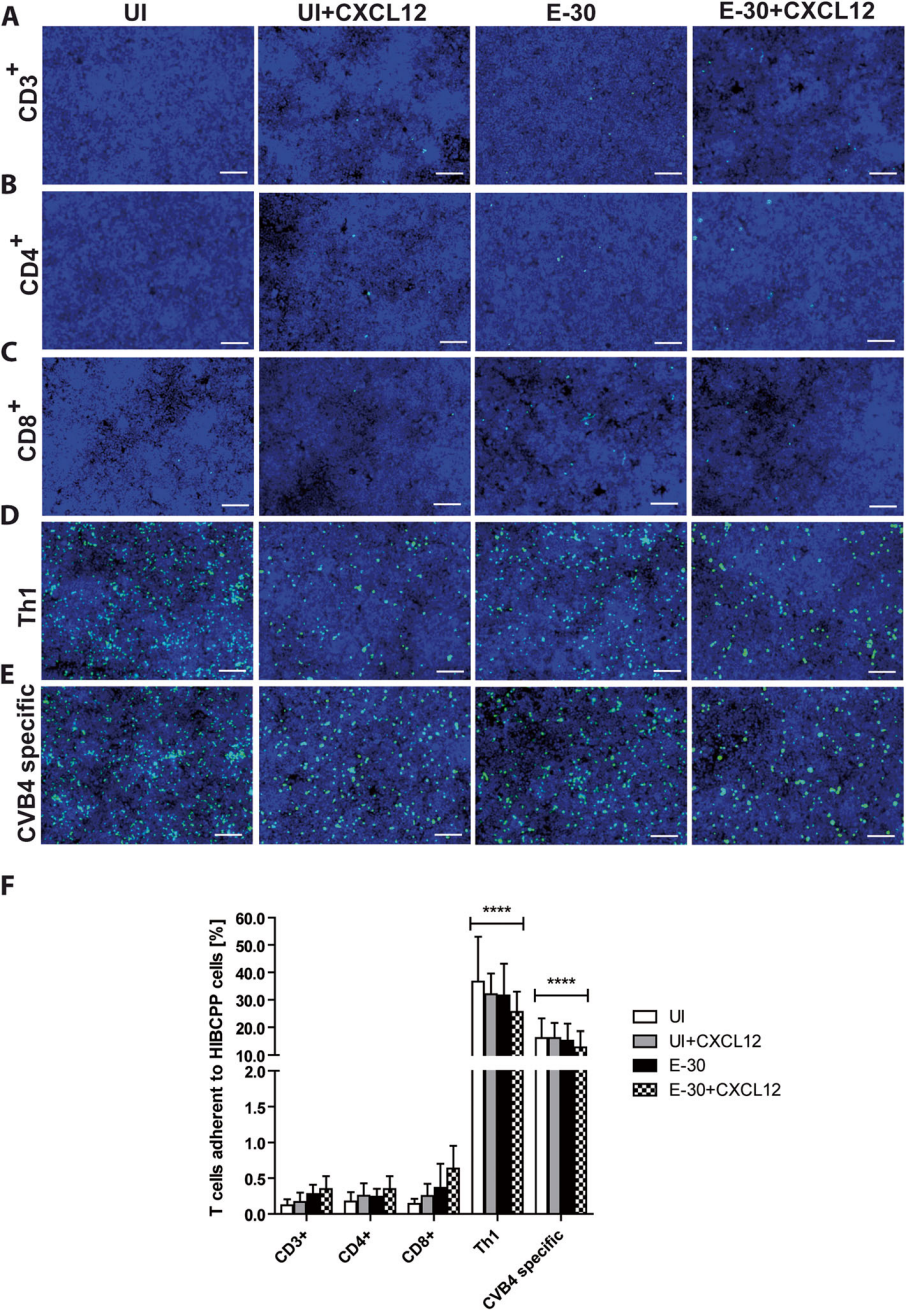
Th1 and CVB4-specific T cells are retained at the basolateral side of HIBCPP cells after of migration. T cell association with HIBCPP cells after 4 h was analyzed by immunofluorescence microscopy. The following conditions were analyzed: UI, UI + CXCL12, E-30, E-30 + CXCL12. **a** shows representative images for CD3^+^, **b** for CD4^+^, **c** for CD8^+^, **d** for Th1, and **e** for CVB4-specific T cells. Per condition, 10 FOV were taken with X63/1.4 objective lens. T cells were stained in cell tracker green Alexa 488 and nucleus in blue (DAPI). **f** Quantification of the T cells associated to the basolateral side of HIBCPP cells after 4 h for the following conditions UI, UI + CXCL12, E-30, E-30 + CXCL12 and the following T cell subsets: CD3^+^, CD4^+^, CD8^+^, Th1, and CVB4-specific T cells are shown. Data are shown as mean ± SD of 6 independent experiments each performed in triplicate. Statistical significance was calculated using a Tukey–Kramer test. *p* values are displayed as follows: *p* < 0.0001 (****). *p* < 0.0001 (****) was reached comparing all the values for each condition from effector T cells and CVB4-specific T cells with the values for each condition from naïve T cells. The white scale bar represents 100 μm

The interaction of immune cells with the choroid plexus is not well known. Following the migration process of T cell subpopulations, we performed immunofluorescence imaging analyzing the cell association of T cells with the HIBCPP cell layer. We observed a higher number of effector Th1 cells and CVB4-specific T cells adherent to the basolateral side of HIBCPP cells for every condition when compared with naive CD3^+^, CD4^+^, and CD8^+^ T cells (Fig. 4). Quantification revealed a substantial amount of Th1 cells (uninfected control (UI), 36.71 ± 16.31%; UI + CXCL12, 32.10 ± 7.53%; E-30, 31.65 ± 11.59%; E-30 + CXCL12, 25.61 ± 4.41%) and CVB4-specific T cells (UI, 16.26 ± 7.06%; UI + CXCL12, 16.22 ± 5.35%; E-30. 15.21 ± 6.14%; E-30 + CXCL12, 12.76 ± 5.85%) adherent to HIBCPP independent from the respective stimulation condition (Fig. 4f). In contrast, we observed a significantly lower amount of naive T cells associated with HIBCPP cells compared with T effector cells with less than 2 ± 0.5% of cells in every condition (*p* > 0.0001) (Fig. 4a, b, c, f).

### Naive and effector T cells have no impact on barrier function of HIBCPP cell layer

**Fig. 5:**
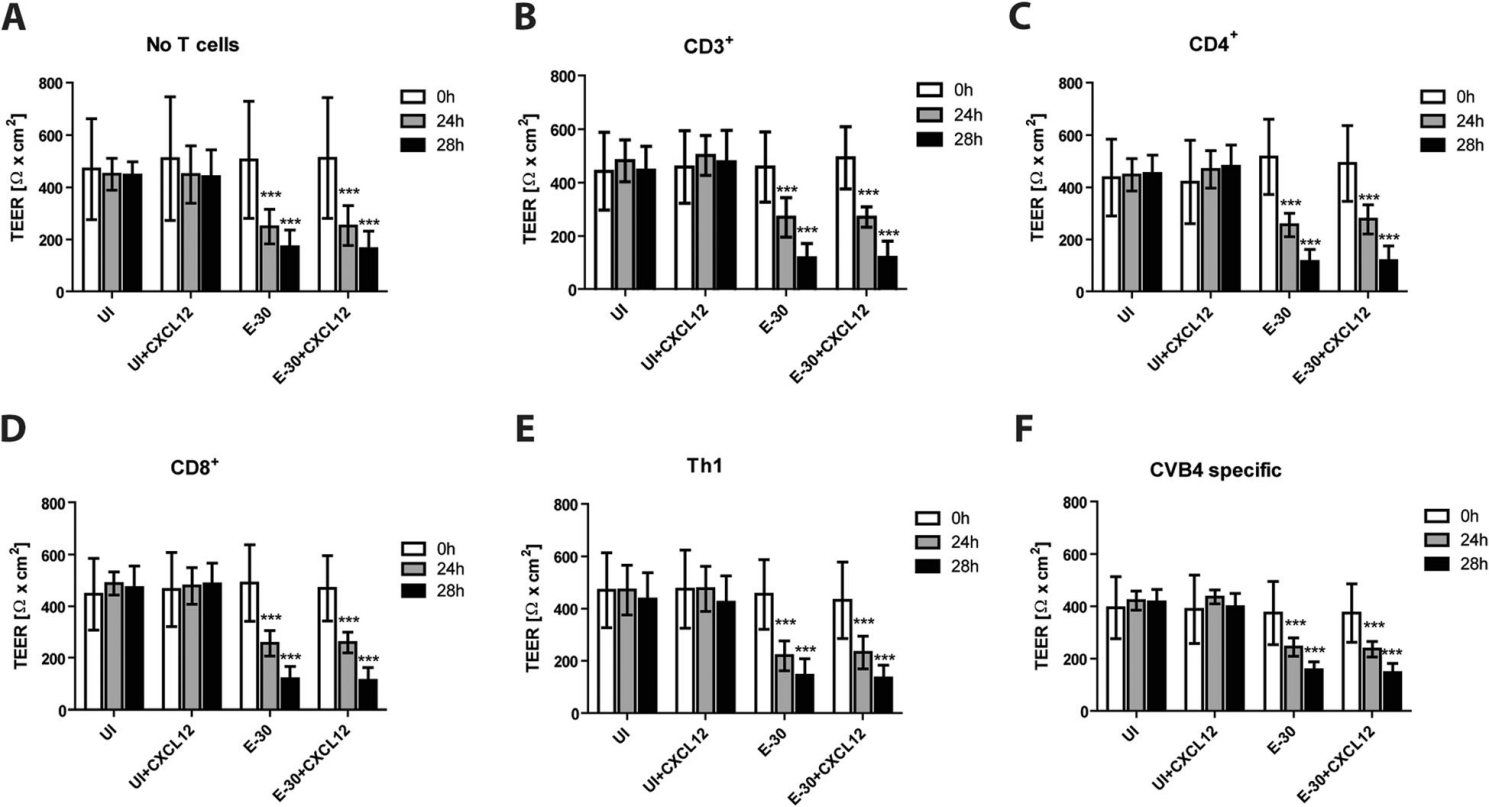
Migration of T cells following E-30 has no impact on TEER of HIBCPP cells. Barrier integrity of HIBCPP cells was evaluated via measurement of the transepithelial resistance (TEER) at T = 0 h (white bars), T = 24 h (gray bars), and T = 28 h (black bars) for every condition uninfected control (UI), UI + CXCL12, E-30, E-30 + CXCL12 and for every cell type. **a** shows the result in the absence of T cells (= no T cells); **b** shows the results with CD3^+^ **c** with CD4^+^, **d** with CD8^+^, **e** with Th1 and **f** with CVB4-specific T cells. Data are shown as mean ± SD of 6 independent experiments each performed in triplicate. Statistical comparisons with *p* values are shown for T = 24 h and T = 28 h compared with the respective UI or UI and apical CXCL12 (=UI + CXCL12) at (T = 0 h). For statistical analysis, a Tukey–Kramer test was used; *p* values are displayed as follows: *p* < 0.0001 (***). *** *p* < 0.0001 was reached comparing the values to the respective control without E-30 infection

A previous study has shown that E-30 infection of HIBCPP cells with a low MOI, such as 0.7, caused alteration of the barrier function without compromising cell viability during the first 28 h of the experiment [31]. Here, we compared the effect of the migration of different T cell subpopulations on HIBCPP barrier function during E-30 infection. We observed a significant decrease in TEER following infection with E-30 at a MOI of 0.7 after 24 h and 28 h (Fig. 5a–f), whereas the TEER in the uninfected conditions (UI and UI + CXCL12) remained stable throughout the experiment. The decrease in TEER was independent of the presence of naive or effector T cells (Additional file 5, Fig. 5a-f) but solely dependent on the infection with E-30. Furthermore, we observed that addition of the chemokine CXCL12 on the apical side of HIBCPP cells did not affect the TEER of the cells.

**Fig. 6:**
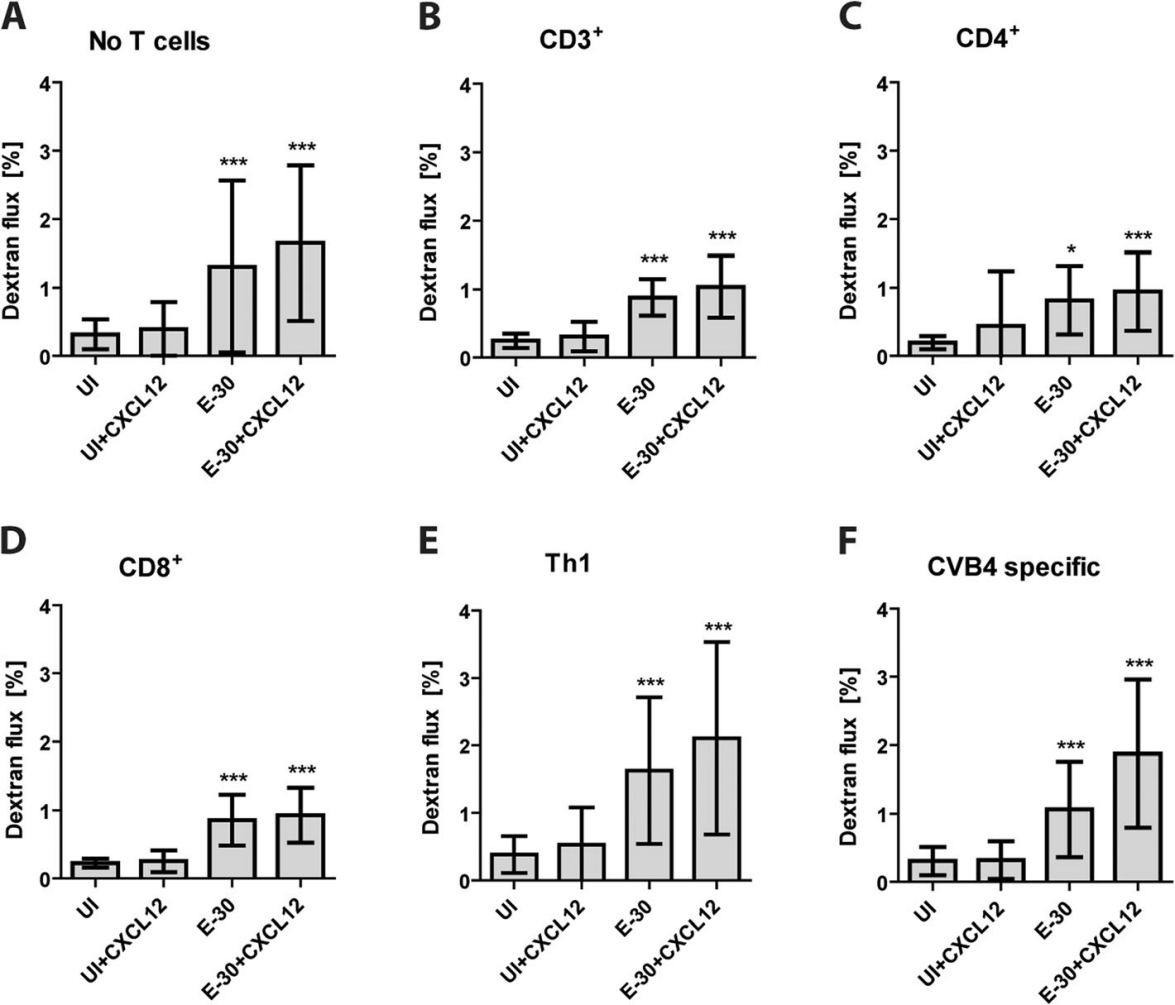
Migration of T cells following E-30 has no impact on the paracellular dextran flux of HIBCPP cells The paracellular flux of the low molecular weight molecule dextran-TexasRed (MW 1000) was measured at the end of the 4-h migration period. Quantification via fluorescent measurement for no T cells (**a**), CD3^+^ (**b**), CD4^+^ (**c**), CD8^+^ (**d**), Th1 (**e**), and for CVB4-specific T cells (f) was performed for each condition (UI, UI + CXCL12, E-30, E-30 + CXCL12). The data are shown as mean ± SD of 6 independent experiments each performed in triplicate. *p* values were obtained comparing the respective conditions in the absence or presence prior to E-30 infection, namely: UI and UI + CXCL12 versus E-30 and E-30 + CXCL12. Statistical significance was calculated using a Tukey–Kramer test. *p* values are displayed as follows: *p < 0.05 and *** *p* < 0.0001. *p < 0.05 and *** *p* < 0.0001 were reached comparing the values to the respective control without E-30 infection

As second parameter of barrier function, the paracellular flux of dextran was evaluated over the 4-h time span of the migration period. We observed a slight, but significant increase in the paracellular flux following infection with E-30 at a MOI of 0.7 (Fig. 6a), whereas the paracellular flux in the uninfected conditions (UI, UI + CXCL12) remained stable throughout the migration experiment. This effect was also independent of the presence of different T cell subpopulations or CXCL12 (Additional file 6, Fig. 6a-f). Nevertheless, the paracellular flux of dextran remained less than 1% per hour in every condition, indicating the maintenance of an adequate high barrier property.

### Naive and effector T cells use paracellular and transcellular pathway migrating through HIBCPP cells

**Fig. 7:**
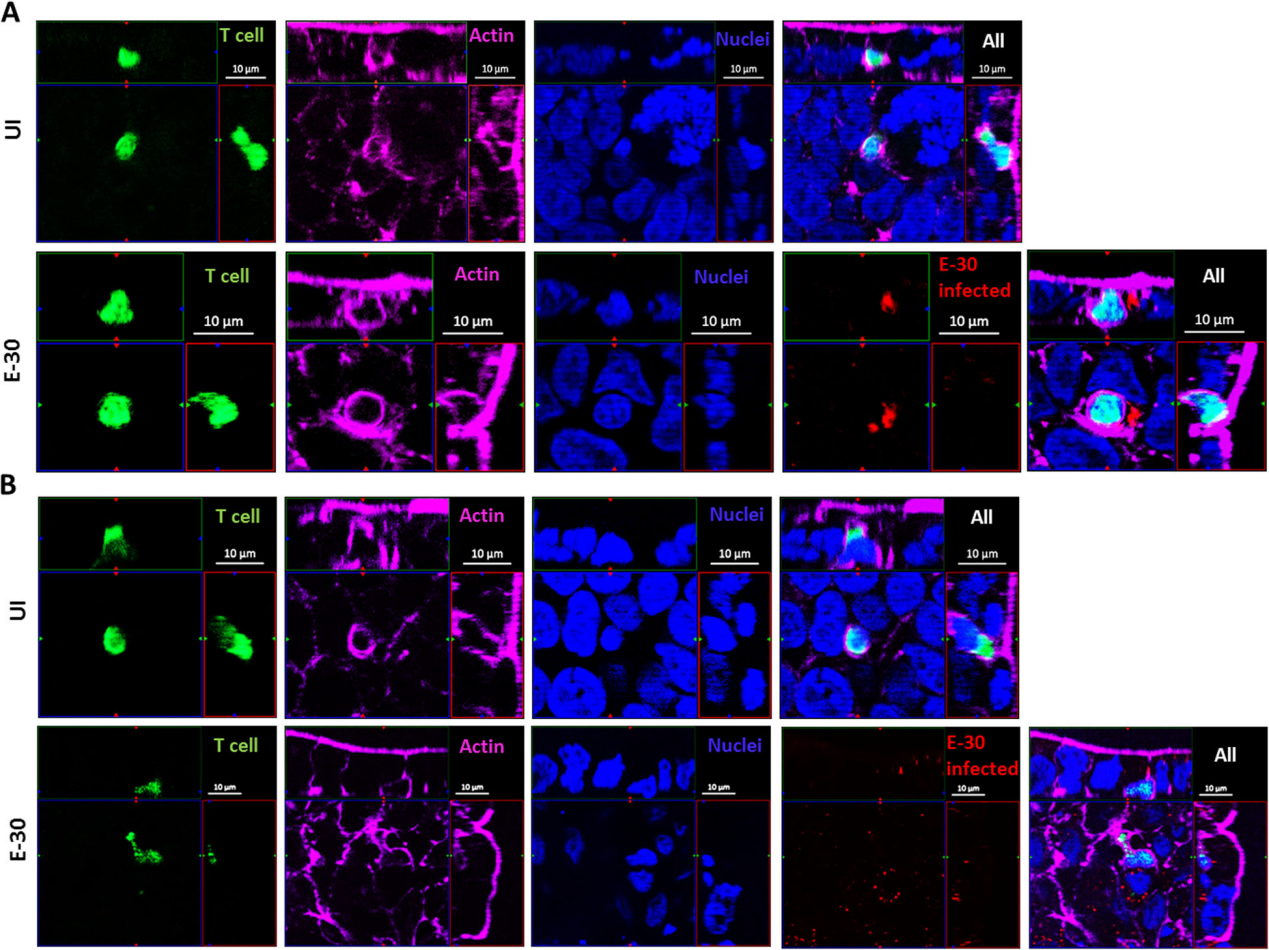
Transcellular and paracellular migration of naive and effector T cells across HIBCPP cells. Immunofluorescence imaging of Th1 T cells performing migration via the transcellular pathway (**a**) and via the paracellular pathway (**b**) across HIBCPP cells in the following conditions: across HIBCPP cells in uninfected and infected with E-30. Images from left to right show T cells stained with cell tracker green (green 488), actin stained with phalloidin (purple 660), nuclei are stained with DAPI (blue 450), and E-30-infected HIBCPP cells are stained with an anti-PAN entero-antibody (red 594). Z-stacks were acquired using Zeiss Apotome and Zen software with X63/1.4 objective lens

Previously, it was shown that CD3^+^ T cells use the paracellular and the transcellular pathway to migrate across the choroid plexus epithelial cells [22, 31]. In this study we performed extensive immunofluorescence analysis of migrating T cell subpopulations through HIBCPP cells. Representative images of Th1 effector cell migration experiments are shown in Fig. 7. We found Th1 effector cells migrating through a HIBCPP cell body in distance to cellular borders (Fig. 7a). Additionally, we observed paracellular migration of Th1 effector cells, squeezing between two adjacent HIBCPP cells, i.e., migrating paracellularly between them (Fig. 7b). All other naive T cell subpopulations and effector T cells were using both the transcellular and paracellular pathway (data not shown).

### T cells adapt their morphology during diapedesis through the HIBCPP cell layer

**Fig. 8:**
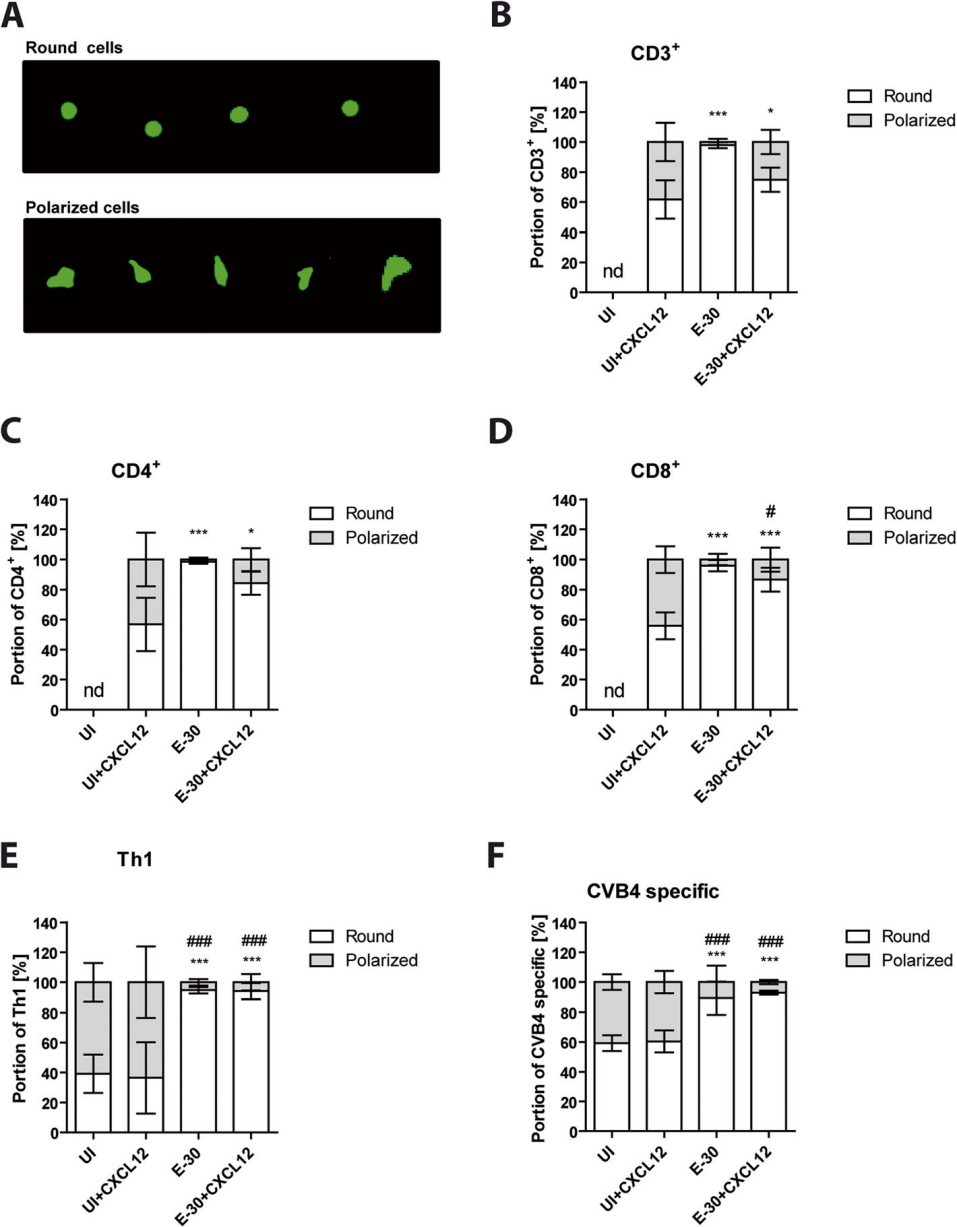
Shape of T cells differs after the migration process depending on the stimulation condition. The shape of the T cells after the migration process from all the T cells subtypes CD3^+^, CD4^+^, CD8^+^, Th1 effector, and CVB4-specific T cells was determined in the following conditions: UI, UI + CXCL12, E-30, E-30 + CXCL12. T cells were labeled in green. The shape of migrated naïve T cells without CXCL12 could not be determined due to low migration rates in uninfected conditions. **a** Immunofluorescence microscopy pictures show “round” (first line) and “polarized” shape of T cells observed 4 h after migration across HIBCPP cells. Quantification of CD3^+^ (**b**), CD4^+^ (**c**), CD8^+^ (**d**), Th1 (**e**), or CVB4-specific T cells (**f**) with a “round” versus “polarized” shape after the migration process. The data are shown as mean ± SD of 6 independent experiments each performed in triplicate. Statistical significance was calculated using a Tukey–Kramer test. *p* values are displayed as follows: *p* < 0.05 (*), *p* < 0.0001 (***), *p* < 0.05 (#), *p* < 0.0001 (###). *p* < 0.0001 (***) were reached comparing UI + CXCL12 to E-30 for all the T cell subsets. *p* < 0.0001 (***) was also reached comparing UI + CXCL12 to E-30 + CXCL12 for CD8^+^, Th1 and CVB4-specific T cells. *p* < 0.05 (*) were reached comparing UI + CXCL12 to E-30 + CXCL12 for CD3^+^ and CD4^+^. *p* < 0.05 (#) was reached comparing E-30 against E-30 + CXCL12 for CD8^+^. *p* < 0.0001 (###) was reached comparing UI and UI + CXCL12 against E-30 and against E-30 + CXCL12, respectively, for Th1 and CVB4-specific T cells

T cells were previously shown to adapt their morphology during the vascular extravasation process [32]. However, T cell morphology and interactions during the migration across the choroid plexus epithelial cells under inflammatory conditions have not yet been investigated. Immunofluorescence imaging revealed that after migration T cells had two distinct phenotypes, which we classified either as “round” or, when a cytoplasmic expansion was present, as “polarized” (Fig. 8a). At the beginning of the abovementioned experimental setup, the naive T cells have a “round” shape and the effector T cells have a “polarized” shape. Next, we quantified the proportion of “round” versus “polarized” T cells following the migration process. In apical presence of CXCL12 the naive T cells showed a higher proportion of “polarized” T cells (38.29 ± 12.82% of CD3^+^, 43.29 ± 17.80% of CD4^+^, and 44.19 ± 8.95% of CD8^+^) when compared with the conditions of E-30-infected HIBCPP cell layers (Fig. 8b–d). Following E-30 infection the vast majority of the migrating naive T cells remained “round,” whereas the CXC12 stimulated T cells, which were mainly “polarized” without infection, acquired a round phenotype. Of naive T cells only a minority were “polarized” after migration through an E-30-infected HIBCPP cell layer (2.06 ± 2.03% of CD3^+^, 1.51 ± 1.40% of CD4^+^, 4.18 ± 3.74% of CD8^+^) (Fig. 8b–d). The morphology of migrated naive T cells without CXCL12 could not be determined and quantified due to low migration rates.

Investigating Th1 effector cells and CVB4-specific T cells we observed in the uninfected control condition even without CXCL12 a high proportion of “polarized” cells, 60.94 ± 12.78% and 40.78 ± 5.20%, respectively (Fig. 8e–f). Moreover, we found approximately the same proportion of “polarized” cells in presence or absence of CXCL12 (Fig. 8e–f). In contrast, after migrating across E-30-infected HIBCPP cells, the majority of the effector T cells were found to have a “round” shape, independent from the presence (94.65 ± 2.18% of Th1 and 89.24.78 ± 11.08% of CVB4-specific T cells) or absence (94.08 ± 5.38% of Th1 and 92.86 ± 1.3% of CVB4-specific T cells) of CXCL12.

### Changes of cell surface expression of adhesion molecules and chemokine receptors after migration of Th1 effector cells through HIBCPP cells

Finally, we investigated the expression of adhesion molecules and chemokine receptors present on Th1 effector cells after the migration process in presence or in absence of E-30 infection by flow cytometry analysis. Additionally, non-migrated Th1 cells were analyzed under the same conditions. Importantly, the majority (95.18 ± 1.66%) of the Th1 effector cells were alive after migrating across the HIBCPP cell monolayer even in presence of E-30 infection (Additional file 7).

**Fig. 9:**
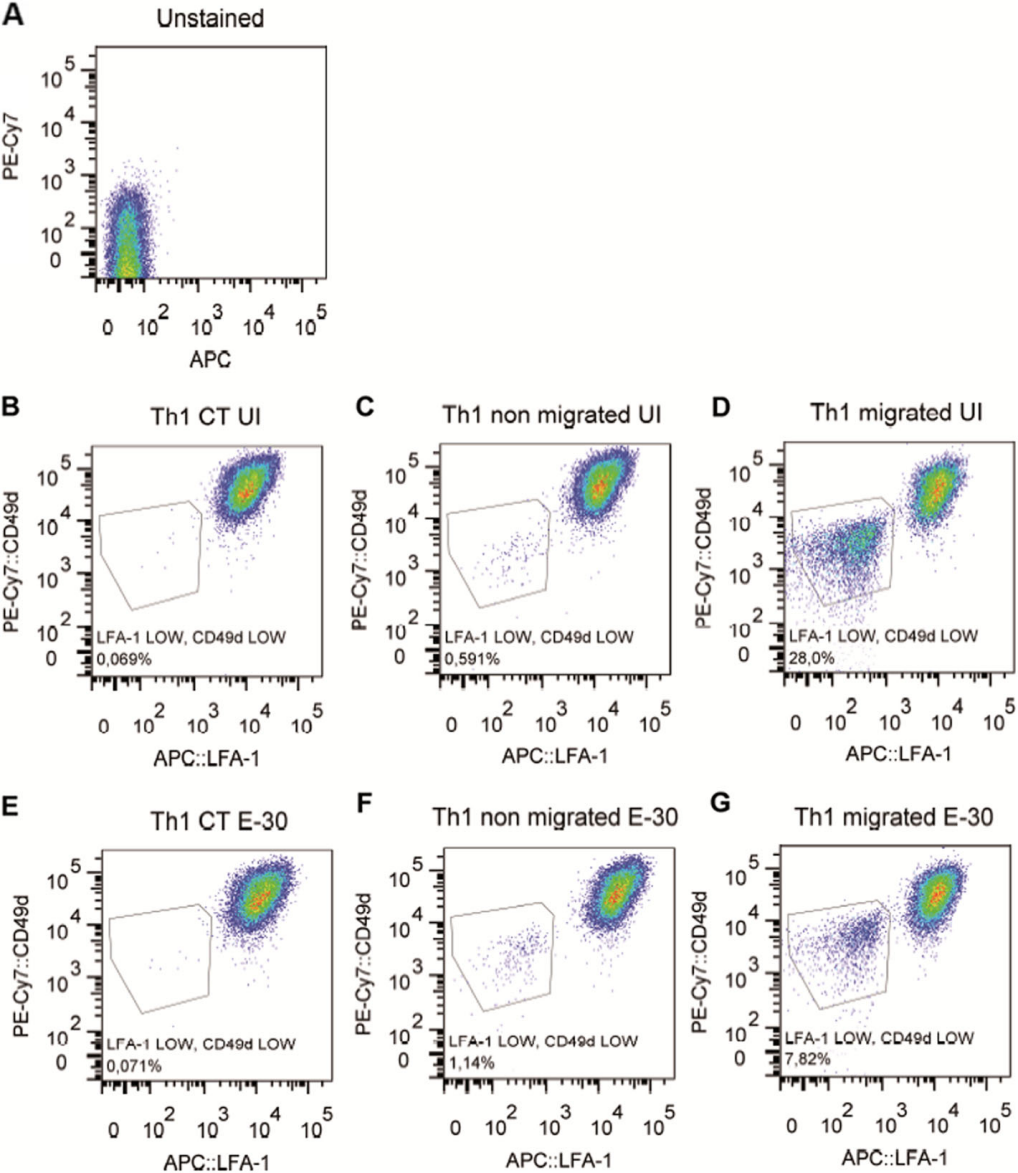
Flow cytometry analyses of LFA-1 and CD49d of Th1 effector cells. LFA-1 and CD49d expression on the surface of Th1 cells was quantified via flow cytometry after 4 h of migration. The dot plots show representative result of the following conditions: **a** unstained Th1 cells, **b** Th1 not in contact with HIBCPP cells in uninfected condition (=Th1 UI CT), **c** Th1 present on the upper compartment of the well in uninfected condition (= UI non migrated Th1), **d** Th1 cells present on the bottom of the well in uninfected condition (= UI migrated Th1), **e** Th1 not in contact with HIBCPP cells in E-30-infected condition (= E-30 CT Th1), **f** Th1 present in the upper compartment of the well in E-30-infected condition (= E-30 non migrated Th1), **g** Th1 cells present on the bottom of the well in E-30-infected condition (= E-30 migrated Th1). Filters (*n* = 36 each) were pooled for each experiment and each condition. Representative data of four independent experiments are shown

**Fig. 10:**
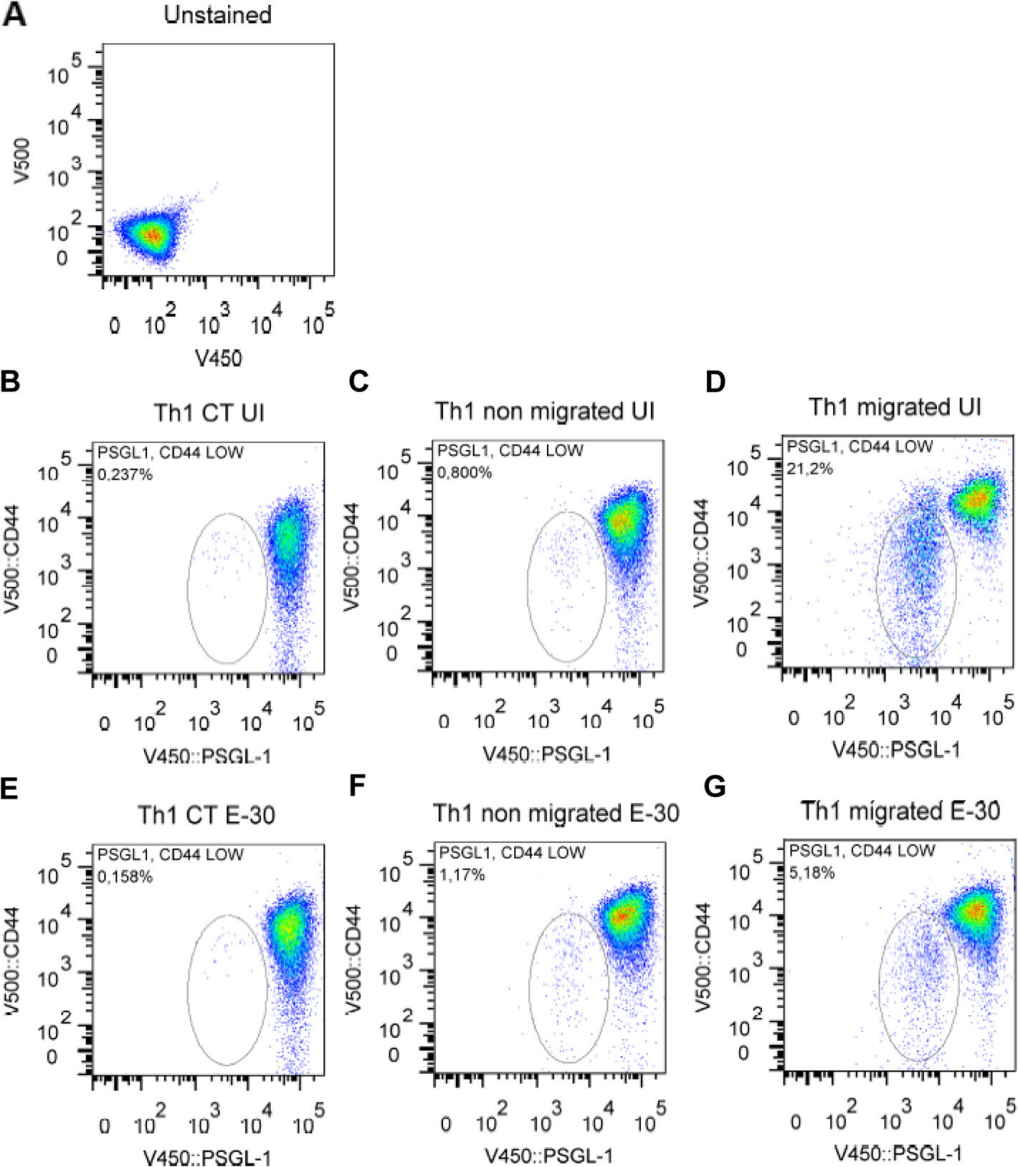
Decreased expressions of CD44 and PSGL-1 on Th1 effector cells following migration across HIBCPP cells. Expression of the receptor CD44 and PSGL-1 present in the Th1 cells was quantified, via flow cytometry measurement after 4 h of migration. The dot plots show representative expression of CD44 and PSGL-1 on Th1 in different conditions: **a** unstained Th 1 cells, **b** Th1 not in contact with HIBCPP cells in uninfected condition (=Th1 CT UI), **c** Th1 present in the upper compartment of the well in uninfected condition (=Th1 non migrated UI), **d** Th1 cells present in the bottom of the well in uninfected condition (= Th1migrated UI), **e** Th1 not in contact with HIBCPP cells in E-30-infected condition (=Th1 CT E-30), **f** Th1 present in the upper compartment of the well in uninfected condition (=Th1 non migrated UI), **g** Th1 cells present on the bottom of the well in E-30-infected condition (= Th1 migrated E-30). Filters (*n* = 36 each) were pooled for each experiment and each condition. Representative data of four independent experiments are shown

After crossing HIBCPP cells two Th1 cell populations were observed. One population displayed a high co-expression of LFA-1/CD49d, whereas the other presented a significantly lower co-expression of LFA-1/CD49d, independently of the presence of E-30 (Fig. 9). The same phenomenon was observed for the co-expression of PSGL-1/CD44 (PSGL; P-selectin glycoprotein ligand-1) (Fig. 10). Furthermore, we analyzed the expression levels of several other of adhesion molecules and chemokine receptors on Th1 effector cells, but identified no significant regulation (β7-integrin, CD62L, CD29, CXCR3, CCR4, CCR6, CCR7) (data not shown).

## Discussion

Echovirus 30 is one of the main NPEV causing extensive outbreaks of meningitis every year worldwide [2, 33]. Clinical studies revealed the presence of high concentrations of T cells and inflammatory cytokines in the CSF of patients with enterovirus infection [8, 11]. Several publications have described the BCSFB as an entry gate for enterovirus into the brain [5]. Previously, a human in vitro model of the BCSFB based on HIBCPP cells has been developed, which allowed us to study migration of immune cells across the BCSFB [20]. To our knowledge this is the first study that investigated the migration of various T cell subpopulations in BCSFB model, also in the context of E-30 infection.

We observed that E-30 infection enhances the migration of CD3^+^, CD4^+^, and CD8^+^ naive T cells across the BCSFB, especially once CXCL12 is apically present. These findings suggest that naive T cells can be highly active in crossing HIBCPP cell layer if they receive activating signals from the environment [34]. Interestingly, when further looking into the subpopulations of naive T cells, we discovered that naive CD8^+^ T cells showed a significantly higher migration rate following E-30 infection, when compared with the other naive T cells. This may be explained by the primary role of CD8^+^ T cells in the clearance of viral infection [35]. The CD8^+^ population has also been shown to constrain and avoid spreading of an infection in the brain [36, 37].

Especially Th1 effector cells were able to effectively cross HIBCPP cells, even in the absence of viral infection. We could demonstrate an enhanced migration of Th1 effector cells across the BCSFB upon infection of HIBCPP cells with E-30 compared with naive cells. Migration of CVB4-specific T cells instead could not further be enhanced by E-30 infection. Thus, we speculate that previous priming of these cells with enterovirus may have prevented an additional stimulatory effect. As for naive T cells we observed the migration of Th1 effector cells was very high in the presence of apical CXCL12, suggesting that these effector cells as naive T cells can also migrate more efficiently if they receive an additional strong activating signal. These data support further the major role of the inflammatory environment in the brain to promote the migration process of T cells into the CNS. In fact, CXCL12 was shown to be upregulated in the CSF of patients with multiple sclerosis or neurological disease, and this was correlated with an increased migration of B cells and T cells into the CNS [38].

Many publications have shown that inflammatory chemokines in the brain were leading to increased migration of immune cells within the brain parenchyma [39, 40]. In one previous publication the secretion of CCL20 by HIBCPP cells following E-30 infection has been demonstrated [31]. Presence of CCL20 in the CSF correlated in vivo with the preferential migration of CD4^+^ T cells expressing high levels of CCR6 into the CNS such as Th1 T cells through the choroid plexus in mice [41]. In a mice model of experimental autoimmune encephalomyelitis (EAE) symptoms were exacerbated due to BCSFB-mediated upregulation of Irgm1, a GTPase protein, increasing the disruption of the BCSFB leading to the infiltration of lymphocytes across the choroid plexus [42]. Moreover, immune cells were shown to enter the brain parenchyma via the BCSFB in a mouse model of ischemic stroke [43].

In our study we further observed in our experimental setup that T cells strongly adhered at the basolateral side of HIBCPP cells without completing transmigration, and this was observed under all stimulatory conditions. To further migrate these immune cells may need to receive specific signals from the environment, which are yet to be identified. This highlights the great interaction potential of effector T cells (both CVB4 and Th1) associating with the BCSFB. In fact, the BCSFB is an important immunological compartment within the CNS orchestrating signals from both, the CNS to the periphery and vice versa [44]. In vivo studies in mice have revealed the choroid plexus as a reservoir for T cells allowing a fast entry into the CNS to rapidly respond to any sign of neuroinflammation, indicating also an extensive communication function of the choroid plexus with the immune cells [34, 45].

Analyzing HIBCPP barrier function after T cell migration we found a decreased TEER after 24 h and 28 h of E-30 infection independently of the presence of naive and effector T cells. Previously, it was shown that E-30 infection of HIBCPP cells affects barrier function via modulation of tight and adherens junctions [17]. The presence of CXCL12, a pro-inflammatory chemokine, had no impact on the properties of the HIBCPP cells. In contrast, loss of cell polarity through CXCL12 has been demonstrated at the BBB. This was shown to lead to leukocyte migration and neuroinflammation (reviewed in [46]).

Next, we investigated the migratory pathways of naive and effector T cells across HIBCPP cells. As previously shown, we could detect both the para- and the transcellular pathway for CD3^+^ naive T cells [31]. Interestingly, also CD4^+^, CD8^+^, and effector T cells were performing migration using both pathways. However, we could not identify a preferentially used pathway for the naive or effector T cells, since quantification of migrated cells in our experimental setup was not possible. Quantification was demonstrated to be feasible in specific cell lines or animals with e.g. GFP labeled tight or adherens junctions as published by others. In mice EAE experiments for example, it was shown that effector T cells were using different pathways depending on the stimulatory environment, such as the level of ICAM adhesion molecules present at the BBB [47]. In previous experiments of our group, PMN were preferentially using the transcellular pathway to migrate through infected primary porcine choroid plexus epithelial cells (PCPEC) infected with *S. suis* rather than the paracellular pathway [48]. Also in an in vitro model of the BBB the same observation was made, where PMN preferentially also used the transcellular pathway to migrate through the barrier [49]. Another in vitro study using rat choroid plexus epithelium inverted cell culture model indicated that T cells predominantly used the paracellular pathway through the BSCFB under inflammatory conditions [50]. Further investigations in different experimental models could be of help to determine the preferential pathway used by the T cells to migrate through the BCSFB.

We further characterized the phenotype of T cells after the migration process and found two distinct shapes, “round” and “polarized.” CXCL12 stimulation induced polarization of naive T cells, whereas a “round” phenotype could be induced by E-30 infection, even in presence of the chemokine CXCL12. In the context of HSV-1 infection it was shown that polarization with the formation of pseudopods in activated T cells was correlated with the enhanced entry of HSV-1, leading to increased production of viral particles within these cells [51]. Still, our data on T cell phenotype are preliminary and future studies on a molecular level should confirm the data in more detail.

Expressions of adhesion molecules and chemokine receptors at the surface of migrating leukocytes are also important factors characterizing the T cell phenotype. In the context of neuroinflammation at the BBB, extensive knowledge exists on the importance of several adhesion molecules for T cell migration, whereas little is known at the BCSFB [52]. To decipher the specific effects of the enteroviral infection, we focused on experiments without CXCL12, since the latter may have potential strong alterations on several integrins. Our investigations showed for the first time in a BCSF barrier model that after migration a high percentage of Th1 effector T cells downregulate PSGL-1 at the surface compared with non-migrated Th1 effector T cells. Previous publications have presented PSGL-1 as an adhesion molecule that mediates especially the step of T cell rolling in T cell migration, both across the BBB [52] and in the gut [53]. Also, a decreased level of PSGL-1 for activated T cells has been found to be a marker for homing and long-lasting survival [54].

We further demonstrated that LFA-1, which is a key player in the crawling step [47, 55], was downregulated after migration of effector Th1 cells compared with non-migrated cells. Along these lines, it has been shown that the levels of ICAM-1 and ICAM-2, both ligands for LFA-1, could influence the migratory pathway of the effector T cells across the BBB [47]. Furthermore, in our experiments the level of CD44 expression at the surface of Th1 effector cells declined after migration compared with non-migrated Th1. CD44 was shown to be involved in selective extravasation of T cells during EAE in mice [56]. Also, CD44 is a selectin ligand and plays a major role in the tethering and rolling process [57]. It was demonstrated that CD44 plays a role in selective extravasation of T cells during EAE in mice [56], but also that deletion of CD44 attenuates the symptom of EAE [58]. The observed decrease of CD44 expression following the migration across HIBCPP cells could thus be an additional contributing factor in our model.

Lastly, a reduced level of CD49d was observed on the surface of migrated Th1 cells across HIBCPP cells compared with non-migrated T cells. CD49d, also named alpha-4, builds a complex with beta-1, which is called VLA-4 and is responsible for the capture of T cells on endothelium [59]. The development of a VLA-4 antagonist and usage in patients with multiple sclerosis and inflammatory bowel disease by the inhibition of T cell migration across the endothelium has led to clinical improvement (reviewed in [60]).

Recently, new HLA and killer cell immunoglobulin-like receptors (KIRs) have been identified may also play a role in the immune cell migration and the susceptibility to develop viral meningitis/encephalitis [61]. In addition to natural killer cells, KIR is expressed by a small subpopulation of T cells. Summarizing these findings, we found important cell adhesion molecules on Th1 effector cells, which were altered during the migration process through a BCSFB in vitro model, similar to observations at the BBB.

A potential limitation of our study is that it was solely performed in in vitro experiments. However, we could proof in several previous publications of our own group that our HIBCPP model can elegantly be used to study the pathogenesis of enteroviral meningitis [20, 21, 31]. Although some mouse studies on the neurovirulence of enteroviruses such as Coxsackie virus B [62] or Enterovirus 71 [63, 64] exist, no validated in vivo model for Echovirus 30, the most frequently isolated pathogen in enteroviral meningitis worldwide, exists so far. Importantly, mouse models for enterovirus infection for example have been discussed to have several limitations such as the requirement for substantial virus adaptation, and immunodeficient or receptor-transgenic mouse strains. Moreover, mouse models do not closely mimic human disease [65].

Therefore, we believe that our new findings can significantly contribute to the understanding of the pathogenesis of enteroviral meningitis. Especially the role of different T cell subpopulations in the pathogenesis of enteroviral CNS infections has never been addressed before and extensively studied in this work.

## Conclusion

Taken together, our results have shown that effector T cells compared with naive T cells have a higher capability to migrate through HIBCPP cells. Following enterovirus infection, and in the absence of apical CXCL12, the migration of Th1 effector cells was significantly enhanced. Moreover, effector T cells remain adherent to HIBCPP to a large extent potentially contributing to immunosurveillance. E-30 infection increased the migration CD8^+^ T cells, indicating at their specific role during viral CNS infection. Further immunofluorescence analysis revealed that all T cell subpopulations were capable to migrate through HIBCPP, using the paracellular as well as transcellular pathway, pointing to a similar migrating pattern for all T cell subpopulations. In the course of migration T cell subpopulations were adapting their phenotype depending on the stimulation environment. We especially observed an alteration of T cell morphology in presence of E-30 infection, which may have consequence on precise T cell phenotype and T cell function but needs further investigation on a molecular level. Moreover, our results suggest that Th1 effector cells use specific adherence molecules during diapedesis through the BCSFB, which are known to be involved during the migration through the BBB, such as PSGL-1, LFA-1, CD44, and CD49d. Further experimental studies are needed to underline these findings.

## Supplementary information

**Additional file 1.** Dot plot of the sorting strategy via flow cytometry of human T cells. Dot plot representing the selection pathway used to determine the various T cells subsets. Th1, Th2 and Th17 were sorted following the expression of their chemokine receptors via FACS. At first naïve cells and memory cells were sorted from the PBMCs via their expression of CCR7 and CD45RA. Naïve cells have a high expression of CD45RA and CCR7 compared to memory cells. Th1 and Th2 have low expression of CCR6 compared to Th17. Th1 cells are CCR4^low^ and CXCR3^high^. Th2 cells are CCR4^high^ and CXCR3^low^, and Th17 are CCR4^high^.

**Additional file 2.** Dot plot of the expression of IL-4 and INF-γ on Th1 effector T cells at the end of the expansion. Dot plot representing the expression of CD45RA, CD4, IL-4 and INF-γ via flow cytometry of the Th1 effector T cells at the end of the expansion. This dot plot is representative of four independent experiments.

**Additional file 3.** Quantification of the viral copies of E-30/filter at 24 h post-infection. At T = 0 h and T = 24 h the HIBCPP cells were rinsed in PBS and further lysed in PBS 5% Triton. The suspension was further centrifuged at 4000 rpm for 15 min at 4 °C. The aliquoted were frozen at − 80 °C. The number of viral copies was determined via Quantitative TaqMan real-time PCR analysis as described in this paper [20]. Representative data of three independent experiments are shown.

**Additional file 4.** Th1 effector T cell single cell selection for the flow cytometry analysis. First, the population of T effector cells was selected using the SSC-A and FSC-A parameter (A). second single cells were selected using FSC-A FSC-W parameter (B) and lastly another single cell selection was performed using SSC-A, SSC-W parameter (C). Representative data of four independent experiments are shown.

**Additional file 5. **Migration of T cells following E-30 has no impact on TEER of HIBCPP cells. The table is showing all - *p*-values.

**Additional file 6.** Migration of T cells following E-30 has no impact on the paracellular dextran flux of HIBCPP cells. The table is showing all - p-values.

**Additional file 7.** Live/dead analysis of Th1 effector T cells after migration through HIBCPP cells. Following the migration, the Th1 effector cells were incubated with a live–dead dye (APC-Cy7). In the following analyses the population of dead Th1 effector T cells was excluded. Representative data of four independent experiments are shown.

## Data Availability

All data generated or analyzed during this study are included in this published article (and its supplementary information files).

## Abbreviations

BBB: Blood–brain barrier
BCSFB: Blood–cerebrospinal fluid barrier
CD3^+^, CD4^+^, CD8^+^: Cluster of differentiation
CNS: Central nervous system
CSF: Cerebrospinal fluid
CVB4: Coxsackievirus B4, family *Picornaviridae*, genus Enterovirus, species Human enterovirus B
EV: Echovirus, family *Picornaviridae*, genus Enterovirus, species Human enterovirus B
FCS: Fetal calf serum
HIBCPP: Human immortalized brain choroid plexus papilloma cells
MAM: Migration assay medium
MOI: Multiplicity of infection
NPEV: Non-polio enterovirus
SD: Standard deviation
T cells: T lymphocytes
TEER: Transepithelial electrical resistance
TJ: Tight junction

## References

[1] Oberste MS, Maher K, Kennett ML, Campbell JJ, Carpenter MS, Schnurr D, Pallansch MA (1999). Molecular epidemiology and genetic diversity of echovirus type 30 (E30): genotypes correlate with temporal dynamics of E30 isolation. J Clin Microbiol.

[2] Holmes CW, Koo SS, Osman H, Wilson S, Xerry J, Gallimore CI, Allen DJ, Tang JW (2016). Predominance of enterovirus B and echovirus 30 as cause of viral meningitis in a UK population. J Clin Virol.

[3] Bouslama L, Gharbi J, Aouni M (2006). Analysis of the genetic and the corresponding antigenic variability of the VP1 3′ end of ECHO virus type 11 and ECHO virus type 30. Virus Genes.

[4] Kieslich M, Acconci D, Berger A, Jarisch A, Bohles H, Bollinger M, Jacobi G, Hernaiz Driever P (2002). Diagnosis and outcome of neurotropic enterovirus infections in childhood. Klin Padiatr.

[5] Huang HI, Shih SR (2015). Neurotropic Enterovirus infections in the central nervous system. Viruses.

[6] Pichichero ME, McLinn S, Rotbart HA, Menegus MA, Cascino M, Reidenberg BE (1998). Clinical and economic impact of enterovirus illness in private pediatric practice. Pediatrics.

[7] Muehlenbachs A, Bhatnagar J, Zaki SR (2015). Tissue tropism, pathology and pathogenesis of enterovirus infection. J Pathol.

[8] Sulik A, Kroten A, Wojtkowska M, Oldak E (2014). Increased levels of cytokines in cerebrospinal fluid of children with aseptic meningitis caused by mumps virus and echovirus 30. Scand J Immunol.

[9] Man S, Tucky B, Cotleur A, Drazba J, Takeshita Y, Ransohoff RM (2012). CXCL12-induced monocyte-endothelial interactions promote lymphocyte transmigration across an in vitro blood-brain barrier. Sci Transl Med.

[10] Rudolph H, Prieto Dernbach R, Walka M, Rey-Hinterkopf P, Melichar V, Muschiol E, Schweitzer-Krantz S, Richter JW, Weiss C, Bottcher S (2017). Comparison of clinical and laboratory characteristics during two major paediatric meningitis outbreaks of echovirus 30 and other non-polio enteroviruses in Germany in 2008 and 2013. Eur J Clin Microbiol Infect Dis.

[11] Ahlbrecht J, Hillebrand LK, Schwenkenbecher P, Ganzenmueller T, Heim A, Wurster U, Stangel M, Suhs KW, Skripuletz T (2018). Cerebrospinal fluid features in adults with enteroviral nervous system infection. Int J Infect Dis.

[12] Li H, Li S, Zheng J, Cai C, Ye B, Yang J, Chen Z (2015). Cerebrospinal fluid Th1/Th2 cytokine profiles in children with enterovirus 71-associated meningoencephalitis. Microbiol Immunol.

[13] Begley DJ (2004). Delivery of therapeutic agents to the central nervous system: the problems and the possibilities. Pharmacol Ther.

[14] DiNunzio JC, Williams RO (2008). CNS disorders--current treatment options and the prospects for advanced therapies. Drug Dev Ind Pharm.

[15] Spector R, Johanson CE (1989). The mammalian choroid plexus. Sci Am.

[16] Ghersi-Egea JF, Strazielle N, Catala M, Silva-Vargas V, Doetsch F, Engelhardt B (2018). Molecular anatomy and functions of the choroidal blood-cerebrospinal fluid barrier in health and disease. Acta Neuropathol.

[17] Wolburg H, Paulus W (2010). Choroid plexus: biology and pathology. Acta Neuropathol.

[18] Redzic ZB, Segal MB (2004). The structure of the choroid plexus and the physiology of the choroid plexus epithelium. Adv Drug Deliv Rev.

[19] Tenenbaum T, Steinmann U, Friedrich C, Berger J, Schwerk C, Schroten H (2013). Culture models to study leukocyte trafficking across the choroid plexus. Fluids Barriers CNS.

[20] Schneider H, Weber CE, Schoeller J, Steinmann U, Borkowski J, Ishikawa H, Findeisen P, Adams O, Doerries R, Schwerk C (2012). Chemotaxis of T-cells after infection of human choroid plexus papilloma cells with echovirus 30 in an in vitro model of the blood-cerebrospinal fluid barrier. Virus Res.

[21] Dahm T, Adams O, Boettcher S, Diedrich S, Morozov V, Hansman G, Fallier-Becker P, Schadler S, Burkhardt CJ, Weiss C (2018). Strain-dependent effects of clinical echovirus 30 outbreak isolates at the blood-CSF barrier. J Neuroinflammation.

[22] Steinmann U, Borkowski J, Wolburg H, Schroppel B, Findeisen P, Weiss C, Ishikawa H, Schwerk C, Schroten H, Tenenbaum T (2013). Transmigration of polymorphnuclear neutrophils and monocytes through the human blood-cerebrospinal fluid barrier after bacterial infection in vitro. J Neuroinflammation.

[23] Borkowski J, Li L, Steinmann U, Quednau N, Stump-Guthier C, Weiss C, Findeisen P, Gretz N, Ishikawa H, Tenenbaum T (2014). Neisseria meningitidis elicits a pro-inflammatory response involving IkappaBzeta in a human blood-cerebrospinal fluid barrier model. J Neuroinflammation.

[24] Korin B, Ben-Shaanan TL, Schiller M, Dubovik T, Azulay-Debby H, Boshnak NT, Koren T, Rolls A (2017). High-dimensional, single-cell characterization of the brain’s immune compartment. Nat Neurosci.

[25] Meeker RB, Williams K, Killebrew DA, Hudson LC (2012). Cell trafficking through the choroid plexus. Cell Adhes Migr.

[26] Schwerk C, Papandreou T, Schuhmann D, Nickol L, Borkowski J, Steinmann U, Quednau N, Stump C, Weiss C, Berger J (2012). Polar invasion and translocation of Neisseria meningitidis and Streptococcus suis in a novel human model of the blood-cerebrospinal fluid barrier. PLoS One.

[27] Sallusto F, Schaerli P, Loetscher P, Schaniel C, Lenig D, Mackay CR, Qin S, Lanzavecchia A (1998). Rapid and coordinated switch in chemokine receptor expression during dendritic cell maturation. Eur J Immunol.

[28] Engen SA, Valen Rukke H, Becattini S, Jarrossay D, Blix IJ, Petersen FC, Sallusto F, Schenck K (2014). The oral commensal Streptococcus mitis shows a mixed memory Th cell signature that is similar to and cross-reactive with Streptococcus pneumoniae. PLoS One.

[29] Mahnke YD, Brodie TM, Sallusto F, Roederer M, Lugli E (2013). The who’s who of T-cell differentiation: human memory T-cell subsets. Eur J Immunol.

[30] Marttila J, Juhela S, Vaarala O, Hyoty H, Roivainen M, Hinkkanen A, Vilja P, Simell O, Ilonen J (2001). Responses of coxsackievirus B4-specific T-cell lines to 2C protein-characterization of epitopes with special reference to the GAD65 homology region. Virology.

[31] Dahm T, Frank F, Adams O, Lindner HA, Ishikawa H, Weiss C, Schwerk C, Schroten H, Tenenbaum T, Rudolph H (2017). Sequential transmigration of polymorphonuclear cells and naive CD3(+) T lymphocytes across the blood-cerebrospinal-fluid barrier in vitro following infection with echovirus 30. Virus Res.

[32] Schlickum S, Sennefelder H, Friedrich M, Harms G, Lohse MJ, Kilshaw P, Schon MP (2008). Integrin alpha E (CD103) beta 7 influences cellular shape and motility in a ligand-dependent fashion. Blood.

[33] Mantadakis E, Pogka V, Voulgari-Kokota A, Tsouvala E, Emmanouil M, Kremastinou J, Chatzimichael A, Mentis A (2013). Echovirus 30 outbreak associated with a high meningitis attack rate in Thrace, Greece. Pediatr Infect Dis J.

[34] Strominger I, Elyahu Y, Berner O, Reckhow J, Mittal K, Nemirovsky A, Monsonego A (2018). The choroid plexus functions as a niche for T-cell stimulation within the central nervous system. Front Immunol.

[35] Kaech SM, Wherry EJ (2007). Heterogeneity and cell-fate decisions in effector and memory CD8+ T cell differentiation during viral infection. Immunity.

[36] Brizic I, Susak B, Arapovic M, Huszthy PC, Hirsl L, Kvestak D, Juranic Lisnic V, Golemac M, Pernjak Pugel E, Tomac J (2018). Brain-resident memory CD8(+) T cells induced by congenital CMV infection prevent brain pathology and virus reactivation. Eur J Immunol.

[37] Bantug GR, Cekinovic D, Bradford R, Koontz T, Jonjic S, Britt WJ (2008). CD8+ T lymphocytes control murine cytomegalovirus replication in the central nervous system of newborn animals. J Immunol.

[38] Krumbholz M, Theil D, Cepok S, Hemmer B, Kivisakk P, Ransohoff RM, Hofbauer M, Farina C, Derfuss T, Hartle C (2006). Chemokines in multiple sclerosis: CXCL12 and CXCL13 up-regulation is differentially linked to CNS immune cell recruitment. Brain.

[39] Sonar SA, Lal G (2017). Differentiation and transmigration of CD4 T cells in Neuroinflammation and autoimmunity. Front Immunol.

[40] Sonar SA, Shaikh S, Joshi N, Atre AN, Lal G (2017). IFN-gamma promotes transendothelial migration of CD4(+) T cells across the blood-brain barrier. Immunol Cell Biol.

[41] Restorick SM, Durant L, Kalra S, Hassan-Smith G, Rathbone E, Douglas MR, Curnow SJ (2017). CCR6(+) Th cells in the cerebrospinal fluid of persons with multiple sclerosis are dominated by pathogenic non-classic Th1 cells and GM-CSF-only-secreting Th cells. Brain Behav Immun.

[42] Wang C, Wang C, Dong H, Wu XM, Wang C, Xia F, Li G, Jia X, He S, Jiang X (2013). Immune-related GTPase Irgm1 exacerbates experimental auto-immune encephalomyelitis by promoting the disruption of blood-brain barrier and blood-cerebrospinal fluid barrier. Mol Immunol.

[43] Llovera G, Benakis C, Enzmann G, Cai R, Arzberger T, Ghasemigharagoz A, Mao X, Malik R, Lazarevic I, Liebscher S (2017). The choroid plexus is a key cerebral invasion route for T cells after stroke. Acta Neuropathol.

[44] Baruch K, Schwartz M (2013). CNS-specific T cells shape brain function via the choroid plexus. Brain Behav Immun.

[45] Fisher Y, Strominger I, Biton S, Nemirovsky A, Baron R, Monsonego A (2014). Th1 polarization of T cells injected into the cerebrospinal fluid induces brain immunosurveillance. J Immunol.

[46] Williams JL, Holman DW, Klein RS (2014). Chemokines in the balance: maintenance of homeostasis and protection at CNS barriers. Front Cell Neurosci.

[47] Abadier M, Haghayegh Jahromi N, Cardoso Alves L, Boscacci R, Vestweber D, Barnum S, Deutsch U, Engelhardt B, Lyck R (2015). Cell surface levels of endothelial ICAM-1 influence the transcellular or paracellular T-cell diapedesis across the blood-brain barrier. Eur J Immunol.

[48] Wewer C, Seibt A, Wolburg H, Greune L, Schmidt MA, Berger J, Galla HJ, Quitsch U, Schwerk C, Schroten H, Tenenbaum T (2011). Transcellular migration of neutrophil granulocytes through the blood-cerebrospinal fluid barrier after infection with Streptococcus suis. J Neuroinflammation.

[49] von Wedel-Parlow M, Schrot S, Lemmen J, Treeratanapiboon L, Wegener J, Galla HJ (2011). Neutrophils cross the BBB primarily on transcellular pathways: An in vitro study. Brain Res.

[50] Strazielle N, Creidy R, Malcus C, Boucraut J, Ghersi-Egea JF (2016). T-lymphocytes traffic into the brain across the blood-CSF barrier: evidence using a reconstituted choroid plexus epithelium. PLoS One.

[51] Bhattarakosol P, Donchai P (2015). One of the mechanisms to increase HSV-1 uptake in HSV-1-infected, activated T lymphocytes is the formation of Filopodia. Intervirology.

[52] Sathiyanadan K, Coisne C, Enzmann G, Deutsch U, Engelhardt B (2014). PSGL-1 and E/P-selectins are essential for T-cell rolling in inflamed CNS microvessels but dispensable for initiation of EAE. Eur J Immunol.

[53] Nunez-Andrade N, Lamana A, Sancho D, Gisbert JP, Gonzalez-Amaro R, Sanchez-Madrid F, Urzainqui A (2011). P-selectin glycoprotein ligand-1 modulates immune inflammatory responses in the enteric lamina propria. J Pathol.

[54] Tinoco R, Otero DC, Takahashi AA, Bradley LM (2017). PSGL-1: a new player in the immune checkpoint landscape. Trends Immunol.

[55] Steiner O, Coisne C, Cecchelli R, Boscacci R, Deutsch U, Engelhardt B, Lyck R (2010). Differential roles for endothelial ICAM-1, ICAM-2, and VCAM-1 in shear-resistant T cell arrest, polarization, and directed crawling on blood-brain barrier endothelium. J Immunol.

[56] Brennan FR, O'Neill JK, Allen SJ, Butter C, Nuki G, Baker D (1999). CD44 is involved in selective leucocyte extravasation during inflammatory central nervous system disease. Immunology.

[57] Zarbock A, Ley K, McEver RP, Hidalgo A (2011). Leukocyte ligands for endothelial selectins: specialized glycoconjugates that mediate rolling and signaling under flow. Blood.

[58] Chitrala KN, Guan HB, Singh NP, Busbee B, Gandy A, Mehrpouya-Bahrami P, Ganewatta MS, Tang CB, Chatterjee S, Nagarkatti P, Nagarkatti M (2017). CD44 deletion leading to attenuation of experimental autoimmune encephalomyelitis results from alterations in gut microbiome in mice. Eur J Immunol.

[59] Greenwood J, Wang Y, Calder VL (1995). Lymphocyte adhesion and transendothelial migration in the central nervous system: the role of LFA-1, ICAM-1, VLA-4 and VCAM-1. Off. Immunology.

[60] Yang GX, Hagmann WK (2003). VLA-4 antagonists: potent inhibitors of lymphocyte migration. Med Res Rev.

[61] Tuttolomondo A, Colomba C, Di Bona D, Casuccio A, Di Raimondo D, Clemente G, Arnao V, Pecoraro R, Ragonese P, Aiello A (2018). HLA and killer cell immunoglobulin-like receptor (KIRs) genotyping in patients with acute viral encephalitis. Oncotarget.

[62] Tabor-Godwin JM, Ruller CM, Bagalso N, An N, Pagarigan RR, Harkins S, Gilbert PE, Kiosses WB, Gude NA, Cornell CT (2010). A novel population of myeloid cells responding to coxsackievirus infection assists in the dissemination of virus within the neonatal CNS. J Neurosci.

[63] Yu P, Bao L, Xu L, Li F, Lv Q, Deng W, Xu Y, Qin C. Neurotropism in vitro and mouse models of severe and mild infection with clinical strains of Enterovirus 71. Viruses. 2017;9. 10.3390/v9110351 PMC5707558 29156632

[64] Wang YF, Yu CK (2014). Animal models of enterovirus 71 infection: applications and limitations. J Biomed Sci.

[65] Lulla V, Dinan AM, Hosmillo M, Chaudhry Y, Sherry L, Irigoyen N, Nayak KM, Stonehouse NJ, Zilbauer M, Goodfellow I, Firth AE (2019). An upstream protein-coding region in enteroviruses modulates virus infection in gut epithelial cells. Nat Microbiol.

