# Supplementary figures and images for: Distinct migratory pattern of naive and effector T cells through the blood–CSF barrier following Echovirus 30 infection
Source: J Neuroinflammation. 2019 Nov 21;16:232. doi: 10.1186/s12974-019-1626-x (PMC6868812; doi:10.1186/s12974-019-1626-x)

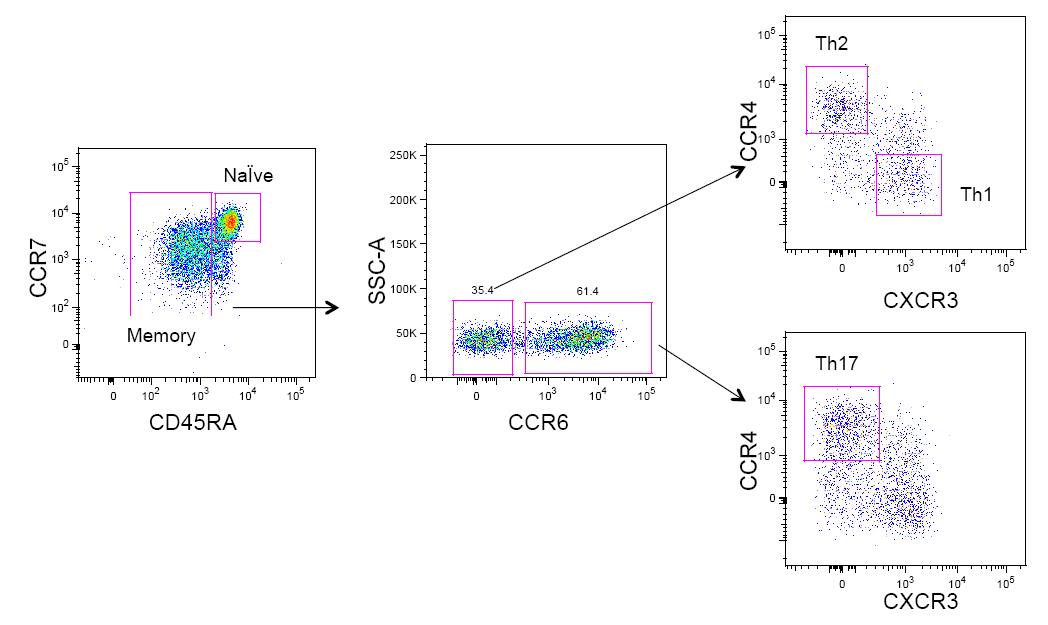

Supplement: Supplementary file 1 — Additional file 1. Dot plot of the sorting strategy via flow cytometry of human T cells. Dot plot representing the selection pathway used to determine the various T cells subsets. Th1, Th2 and Th17 were sorted following the expression of their chemokine receptors via FACS. At first naïve cells and memory cells were sorted from the PBMCs via their expression of CCR7 and CD45RA. Naïve cells have a high expression of CD45RA and CCR7 compared to memory cells. Th1 and Th2 have low expression of CCR6 compared to Th17. Th1 cells are CCR4low and CXCR3high. Th2 cells are CCR4high and CXCR3low, and Th17 are CCR4high. [file 12974_2019_1626_MOESM1_ESM.jpg]

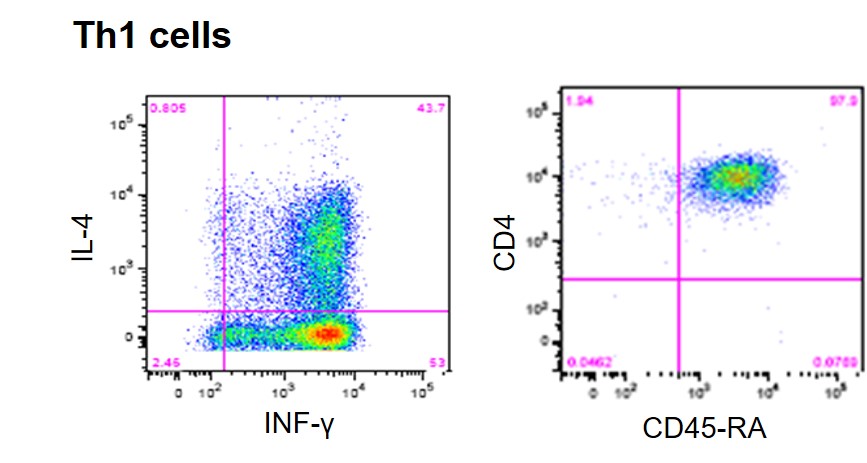

Supplement: Supplementary file 2 — Additional file 2. Dot plot of the expression of IL-4 and INF-γ on Th1 effector T cells at the end of the expansion. Dot plot representing the expression of CD45RA, CD4, IL-4 and INF-γ via flow cytometry of the Th1 effector T cells at the end of the expansion. This dot plot is representative of four independent experiments. [file 12974_2019_1626_MOESM2_ESM.jpg]

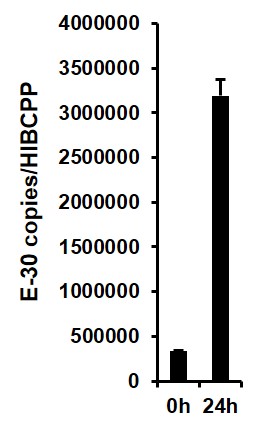

Supplement: Supplementary file 3 — Additional file 3. Quantification of the viral copies of E-30/filter at 24 h post-infection. At T = 0 h and T = 24 h the HIBCPP cells were rinsed in PBS and further lysed in PBS 5% Triton. The suspension was further centrifuged at 4000 rpm for 15 min at 4 °C. The aliquoted were frozen at − 80 °C. The number of viral copies was determined via Quantitative TaqMan real-time PCR analysis as described in this paper [20]. Representative data of three independent experiments are shown. [file 12974_2019_1626_MOESM3_ESM.jpg]

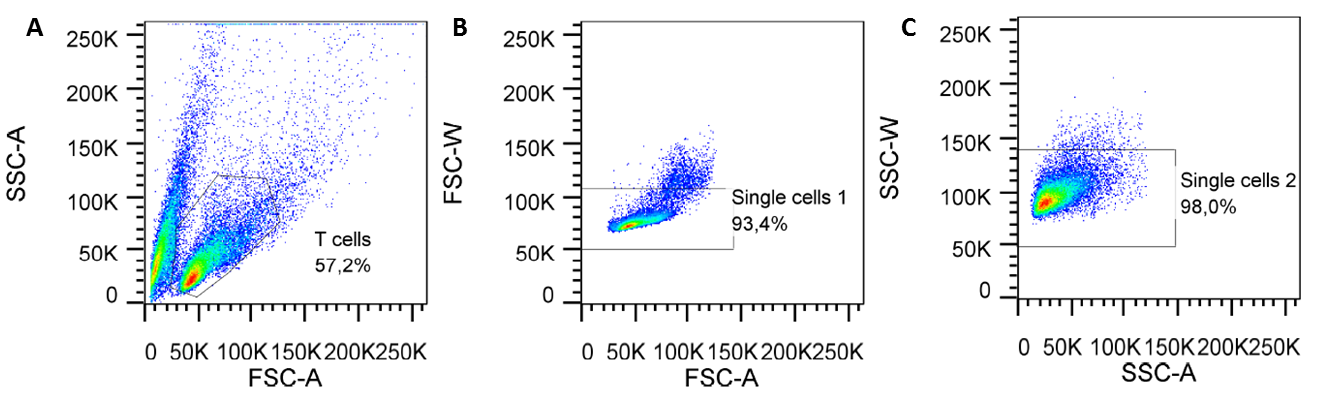

Supplement: Supplementary file 4 — Additional file 4. Th1 effector T cell single cell selection for the flow cytometry analysis. First, the population of T effector cells was selected using the SSC-A and FSC-A parameter (A). second single cells were selected using FSC-A FSC-W parameter (B) and lastly another single cell selection was performed using SSC-A, SSC-W parameter (C). Representative data of four independent experiments are shown. [file 12974_2019_1626_MOESM4_ESM.tif]

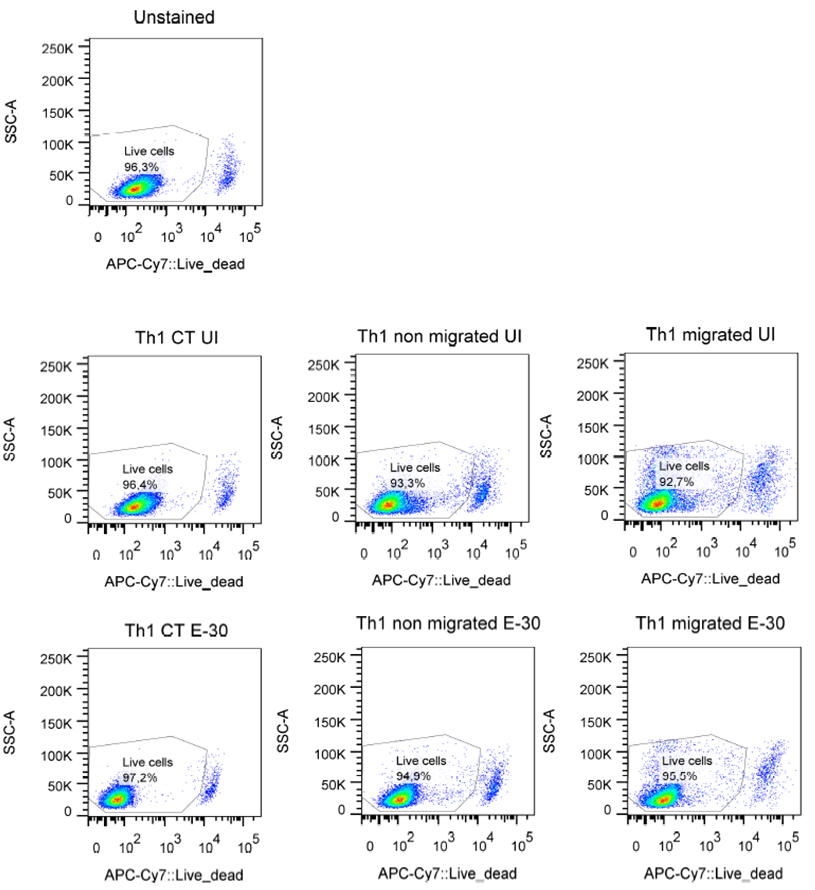

Supplement: Supplementary file 7 — Additional file 7. Live/dead analysis of Th1 effector T cells after migration through HIBCPP cells. Following the migration, the Th1 effector cells were incubated with a live–dead dye (APC-Cy7). In the following analyses the population of dead Th1 effector T cells was excluded. Representative data of four independent experiments are shown. [file 12974_2019_1626_MOESM7_ESM.tif]
